# Imatinib Treatment Causes Substantial Transcriptional Changes in Adult *Schistosoma mansoni In Vitro* Exhibiting Pleiotropic Effects

**DOI:** 10.1371/journal.pntd.0002923

**Published:** 2014-06-12

**Authors:** Christin Buro, Svenja Beckmann, Katia C. Oliveira, Colette Dissous, Katia Cailliau, Richard J. Marhöfer, Paul M. Selzer, Sergio Verjovski-Almeida, Christoph G. Grevelding

**Affiliations:** 1 BFS, Institute for Parasitology, Justus-Liebig-University, Giessen, Germany; 2 Departamento de Bioquímica, Instituto de Química, Universidade de São Paulo, São Paulo, São Paulo, Brasil; 3 CIIL - Center of Infection and Immunity of Lille, Université Lille Nord de France, Inserm U1019, CNRS-UMR 8204, Institut Pasteur de Lille, Lille, France; 4 Laboratoire de Régulation des Signaux de Division, Université Lille 1 Sciences et Technology, EA 4479, IFR 147, Villeneuve d'Ascq, France; 5 MSD Animal Health Innovation GmbH, Molecular Discovery Sciences, Schwabenheim, Germany; University of Melbourne, Australia

## Abstract

**Background:**

Schistosome parasites cause schistosomiasis, one of the most important infectious diseases worldwide. For decades Praziquantel (PZQ) is the only drug widely used for controlling schistosomiasis. The absence of a vaccine and fear of PZQ resistance have motivated the search for alternatives. Studies on protein kinases (PKs) demonstrated their importance for diverse physiological processes in schistosomes. Among others two Abl tyrosine kinases, SmAbl1 and SmAbl2, were identified in *Schistosoma mansoni* and shown to be transcribed in the gonads and the gastrodermis. SmAbl1 activity was blocked by Imatinib, a known Abl-TK inhibitor used in human cancer therapy (Gleevec/Glivec). Imatinib exhibited dramatic effects on the morphology and physiology of adult schistosomes *in vitro* causing the death of the parasites.

**Methodology/Principal Findings:**

Here we show modeling data supporting the targeting of SmAbl1/2 by Imatinib. A biochemical assay confirmed that SmAbl2 activity is also inhibited by Imatinib. Microarray analyses and qRT-PCR experiments were done to unravel transcriptional processes influenced by Imatinib in adult schistosomes *in vitro* demonstrating a wide influence on worm physiology. Surface-, muscle-, gut and gonad-associated processes were affected as evidenced by the differential transcription of e.g. the gynecophoral canal protein gene GCP, paramyosin, titin, hemoglobinase, and cathepsins. Furthermore, transcript levels of VAL-7 and egg formation-associated genes such as tyrosinase 1, p14, and fs800-like were affected as well as those of signaling genes including a ribosomal protein S6 kinase and a glutamate receptor. Finally, a comparative *in silico* analysis of the obtained microarray data sets and previous data analyzing the effect of a TGFβR1 inhibitor on transcription provided first evidence for an association of TGFβ and Abl kinase signaling. Among others GCP and egg formation-associated genes were identified as common targets.

**Conclusions/Significance:**

The data affirm broad negative effects of Imatinib on worm physiology substantiating the role of PKs as interesting targets.

## Author Summary

Schistosomiasis is an infectious disease caused by schistosome parasites, affecting millions of people worldwide. The pathogenic consequences of schistosomiasis are caused by the eggs inducing severe organ inflammations. Praziquantel is widely used to treat schistosomiasis; however, there is fear of resistance developing. Research in the last decades has provided strong evidence for the importance of protein kinases controlling physiological processes in schistosomes. Two Abl-kinases were discovered, whose activities are blocked by Imatinib, an inhibitor known as Gleevec/Glivec from human cancer therapy. *In vitro*, Imatinib treatment led to dramatic effects on morphology and physiology and to the death of adult schistosomes. Besides modeling of the schistosome Abl-kinases we investigated the effect of Imatinib on gene expression in adult *S. mansoni* by performing transcriptomics and discovered a wide influence on the transcription of genes involved in surface-, muscle-, gut- and gonad-associated processes. Comparative *in silico* analyses with data from a previous study indicated a yet unknown association of TGFβ and Abl-kinase signaling in schistosomes. Among others the gynecophoral canal protein gene GCP was identified as a common target. The data obtained demonstrate a substantial influence of Imatinib on physiological processes in adult schistosomes supporting the role of protein kinases as interesting targets.

## Introduction

Schistosomiasis is an infectious disease of worldwide importance caused by parasitic platyhelminthes of the class trematoda such as *Schistosoma haematobium, S. intercalatum, S. japonicum, S. mansoni*, or *S. mekongi*. About 780 million people are at risk of schistosomiasis, and more than 240 million infections emerge annually requiring treatment [1], [2]. Adult schistosomes live in the abdominal veins of their vertebrate hosts. Only if paired, females produce eggs, half of which reach the gut lumen (e.g. *S. mansoni*) or the bladder (*S. haematobium*), to be transported to the environment for continuing the life-cycle. Gut invasion is accompanied by inflammatory processes. The remaining eggs migrate through the blood stream and become trapped in spleen and liver tissue, where granulomas are formed and fibrosis occurs, leading to hepatosplenomegaly and liver cirrhosis [3], [4]. The disease has a high socioeconomic impact causing annual losses of 1.7 to 4.5 million disability adjusted life years (DALYs) of humans living in endemic areas [5], [6], but tourists and travelers can also be affected [7]. Besides humans, animals including cattle can get infected, too, which causes economic losses [8]–[11].

Since there is no vaccine available yet, the main strategy to control schistosomiasis is the regular use of drugs, of which three have the potency to kill schistosomes. Of the drugs available today Metrifonate is active against *S. haematobium* only and Oxamniquine is active against *S. mansoni* only. In contrast to these limitations, although its effectiveness against immature stages is limited Praziquantel (PZQ) is effective against all important schistosome species mainly affecting adults [12]. This and its low price have promoted PZQ as the drug of choice, which is also used in large-scale treatment programs today [13], [14]. However, drug resistance has been recognized as a potential problem since several studies demonstrated PZQ resistance to be inducible in laboratory settings, and field studies provided first indications for the possibility of reduced PZQ efficacy [15]–[18]. Furthermore, multidrug transporters were discovered in schistosomes, of which one was shown to respond to a PZQ challenge [19]. With respect to these facts it is commonly accepted that new drugs are required urgently. To this end research on signal transduction processes in *S. mansoni* has opened new perspectives.

Protein kinases (PKs) are highly conserved signal transduction molecules in the animal kingdom and known to be involved in diverse biological processes such as cell growth and differentiation [20]. Thus PK deregulation can lead to cancer development [21]–[22]. This prompted the search for inhibitors, and meanwhile a number of anticancer drugs targeting PKs are approved for use in humans [21]–[24]. Different studies elucidating principles of schistosome development have shown that PKs play important roles during parasite development [25]–[30]. Due to this, and to the fact that schistosomes can be kept in culture, providing access to adults *ex vivo*, several studies were conducted to investigate whether anticancer drugs would negatively affect PK-controlled processes in schistosomes and cause phenotypic consequences in adults. Indeed, targeting a variety of different kinases using different drugs with PK-inhibiting activities not only showed a negative influence on the reproductive biology of parasites but also remarkable effects on other physiological processes and/or survival *in vitro* [29]–[37].

Among the PKs studied in more detail were protein tyrosine kinases (PTKs) such as Abl kinases, three of which exist in *S. mansoni*. SmAbl1 and SmAbl2 exhibit high sequence similarities to conserved Abl-kinases [38], whereas SmTK6 revealed a Src/Abl hybrid character that was confirmed by structural and biochemical studies [39]. Imatinib was used as an inhibitor to analyze Abl-PK activities in adult *S. mansoni* [38], [39]. Also known as Glivec (Gleevec; Novartis) Imatinib is a small-molecule inhibitor acting as a competitive antagonist of adenosine triphosphate (ATP) binding to Abl-PK, which is applied successfully in human cancer therapy [40]. Structural analyses revealed that the *S. mansoni* Abl-PKs possess the majority of amino acid residues known from studies with the human Abl-kinase to interact with Imatinib [38], [41]. Furthermore, the *Xenopus* oocyte system was shown to be suitable to test the catalytic activity of schistosome tyrosine kinases (TKs) [31], [39]. Thus it was demonstrated that SmAbl1-TK, SmTK6-TK, and SmTK3-TK were able to induce 100% germinal vesicle breakdown (GVBD) [39]. Using competitive GVBD assays it was further demonstrated that Imatinib negatively influenced the kinase activities of SmAbl1-TK (0% GVBD at 1 µM) and SmTK6-TK (0% GVBD at 100 µM). Although the latter required a 100-fold higher concentration compared to SmAbl1-TK, this was explained by the unusual Src/Abl hybrid character of SmTK6. Herbimycin A (Herb A), a Src-TK inhibitor, was not able to fully reduce the GVBD-inducing activity of SmAbl1 (60% GVDB at 10 µM) in contrast to SmTK6, whose activity was fully suppressed at this concentration. The enzymatic activity of SmTK3, a Src kinase used as control, was fully suppressed by Herb A (0% GVBD at 0.01 µM) but revealed nearly no decrease under the influence of Imatinib (still 90% GVBD at 100 µM), confirming the specificity of these inhibitors [39].

Treating adult schistosomes with Imatinib *in vitro* led to dose- and time-dependent effects such as reduced pairing stability, the occurrence of bulges and swellings along the body and, finally, the death of the worms. Microscopic analyses showed not only morphological changes within the gonads of both genders, which appeared disordered, defective in differentiation, and in part apoptotic, but also a detachment and degradation of the gastrodermis. Its complete collapse explained the observed death of the parasites [38]. In a follow-up study it was shown that Dasatinib and Nilotinib, second generation Abl-PK inhibitors of high selectivity, were less effective compared to Imatinib in causing severe or even lethal effects on adult schistosomes *in vitro*. Since Dasatinib and Nilotinib were designed to be even more specific for mutated forms of the human Abl-PK, it was concluded that the more specialized the inhibitor for the human kinase is, the more efficacy for the schistosome kinase gets lost [42].

Because *in situ* hybridizations detected various regions within adult *S. mansoni* where these Abl- and Abl-like PKs of *S. manso*ni were transcribed [36], [38], pointing to different physiological functions these kinases may be involved in, we were interested in investigating the effects of Imatinib at the gene transcription level in treated schistosomes. Besides confirming that Imatinib also affected SmAbl2 kinase activity in a heterologous test system, a transcriptome study was performed by microarray analyses and qRT-PCR verification experiments. Strong evidence was obtained that gene transcription is widely influenced supporting the view that a variety of physiological processes have been affected by this Abl-PK inhibitor. This is in line with previous hypotheses suggesting that PKs, due to their pleiotropic and fundamental roles for schistosome biology, are substantiated targets for novel strategies to treat schistosomiasis [42], [43]. Furthermore, first evidence was obtained that Abl-kinase activities could be part of/or associated with transforming growth factor β (TGFβ) signaling in schistosomes.

## Materials and Methods

### Ethics statement

Experiments with hamsters were performed in accordance with the European Convention for the Protection of Vertebrate Animals used for Experimental and other Scientific Purposes (ETS No 123; revised Appendix A) and were approved by the Regional Council (Regierungspraesidium) Giessen (V54-19 c 20/15 c GI 18/10).

### Parasite stock

*Biomphalaria glabrata* as intermediate snail-host, and hamsters (*Mesocricetus auratus*) as final host were used to maintain the parasite cycle of *S. mansoni* [44]. Adult worms were isolated from hamsters by hepatoportal perfusion 42 days post infection.

### Homology modeling

Chemical Computing Group's MOE 2011 (http://www.chemcomp.com/) molecular modeling suite was used for the homology modeling. The highly conserved catalytic tyrosine domains [38] of human Abl1 (2HYY) and Abl2 (3GVU) were used as templates for the model building of SmAbl1 and SmAbl2, respectively. The two homologous sequences were aligned using MOE's kinase constraints, and the models were built using the Amber99 force field with R-Field solvation. Crystallographic water molecules in an H-bond network with the ligand Imatinib and the ligand itself were used as “Environment for Induced Fit” during model building. Intermediates were refined to medium using the GB/VI scoring, while the final model was refined to Fine with an RMS Gradient of 0.5.

### Molecular Docking

Molecular Docking was carried out using Cambridge Crystallographic Data Centre's GOLD suite 2.5 (http://www.ccdc.cam.ac.uk/). Imatinib was sketched in 2D and converted to 3D using Molecular Networks' CORINA (http://www.molecular-networks.com/). Docking was performed using the Chemscore scoring function with kinase parameters, and the binding site was defined using the position of the Imatinib ligand as modeled in the SmAbl1 and SmAbl2 homology models. Parameters for the genetic algorithm were set to auto. Water molecules used for the model building were allowed to participate in the docking.

### GVBD assays in *Xenopus* oocytes

Sequences of the tyrosine kinase (TK) domains of SmAbl1, SmAbl2, SmTK6, and SmTK3 were obtained by PCR amplification (primers used are given in Supplementary data S1) and cloned into the plasmid pcDNA3.1B (Invitrogen), which contained a T7 promoter for *in vitro* transcription. The resulting constructs SmAbl1-TK, SmAbl2-TK, SmTK6-TK, and SmTK3-TK were sequenced confirming intact open reading frames (ORFs). The plasmid constructs were linearized by *Pme*I and cRNAs were generated using the T7 mMessage mMachine Kit (Ambion, USA). This way capped messenger RNAs (cRNAs) were synthesized *in vitro* and analyzed as previously described [31], [38], before they were injected into stage VI oocytes of *Xenopus laevis*. To this end each oocyte was injected with 60 ng cRNA in the equatorial plane, followed by incubation in ND96 medium at 19°C. GVBD (germinal vesicle breakdown) activity was determined according to the appearance of a white spot at the animal pole 18 h following injections. As shown before, this system was used successfully to monitor schistosome kinase activities [31], [38], [39]. Here it was used to investigate kinase activities under the influence of Imatinib (Enzo Life Sciences; 170 mM stock solution in water), an Abl-kinase inhibitor, or the Src kinase inhibitor Herbimycin A (Herb A) as control (Tocris Bioscience; 10 mM stock solution in DMSO). Pools of 10 oocytes each were injected with SmAbl1-TK, SmAbl2-TK, SmTK3-TK, or SmTK6-TK cRNA and placed in ND96 containing different concentrations of Imatinib (0.01 µM to 100 µM final) or Herb A (0.0001 µM to 10 µM final). As negative control, non-injected oocytes were used. As positive controls, oocytes were incubated with the natural hormonal stimulus progesterone leading to 100% GVBD without further manipulation [45].

### Schistosome *in vitro*-culture and inhibitor treatments

Perfusion was done with M199 medium (Gibco). Paired adult worms were collected using fine tweezers and washed with M199 medium (2x). Subsequently, they were maintained in culture in M199 supplemented with FCS (Gibco; 10%), HEPES (Sigma; 1 M, 1%), and antibiotic/antimycotic mixture (Sigma; 1%) at 37°C and 5% CO_2_ [36], [38]. Inhibitor treatment was performed for 24 h or 48 h with 50 µM Imatinib (Imatinib mesylate, C_29_H_31_N_7_O·CH_3_SO_3_H, dissolved in water; Enzo Life Sciences) as previously described [38]. Control couples were kept in culture for 24 h or 48 h without inhibitor addition but otherwise treated using the same conditions. During the treatment periods, pairing stability and vitality were checked regularly. We defined pairing stability of couples when males kept their female partners within the gynecophoral canal while being sucked with their ventral suckers to the Petri dish. When couples separated, or when males stopped sucking to the Petri dish and/or lay on the side (a sign of decreasing vitality), the appropriate worms were not used for experiments and removed. After completion of treatment, the couples (inhibitor-treated and control) were carefully separated using featherweight tweezers, immediately shock-frozen in liquid nitrogen, and stored at −80°C.

### RNA isolation and microarray experiments

Trizol (Invitrogen) was used to extract RNA from treated or control worms (combined sexes in both cases) followed by a DNAse digestion (RNAeasy kit; Qiagen). The quality of RNA was checked by microfluidic electrophoresis (Bioanalyzer; Agilent Technologies). For microarray experiments a *S. mansoni* custom-designed oligonucleotide platform (60-mers) was used containing 44,000 probes representing nearly the complete *S. mansoni* and *S. japonicum* transcriptome (Agilent Technologies; [46], [47]). All associated information (probes, annotation) is available at Gene Expression Omnibus (GEO) under the accession number GPL8606.

For the microarray experiments RNA from treated and control males and females (300 ng each for biological replicates) was used for cDNA amplification followed by Cy3 and Cy5 labelling during *in vitro* transcription (Quick Amp Labelling Kit, two colors; Agilent Technologies). Dye-swap approaches were done as internal technical replications for each sample. Three microarray hybridizations were performed for each sample (inhibitor treatment for 24 and 48 h as well as control), which included two technical replicates for each of the three biological replicates. As probe for hybridization, 825 ng cRNA of each labelled inhibitor sample was used and combined with a control sample labelled with the opposite dye. Hybridization was done at 65°C for 17 h with rotation followed by slide washing (according to the Agilent manual) and scanning (Gene Pix 4000B Scanner; Molecular Devices). Raw data were acquired using the Feature Extraction software (Agilent Technologies). They are available under GEO study number GSE53154. For subsequent data analyses, genes were considered as transcribed only if the corresponding probe had a signal significantly higher than background (using default parameters from the Feature Extraction software and considering the “IsPosAndSig” result from the output). In addition, signals of a probe had to occur in at least 75% of all replicates and in at least one of the two conditions (inhibitor-treated or control) independent of the length of cultivation (24/48 h treatment versus 24/48 h untreated control). The quality of the microarray expression data was assessed by the overall Pearson correlation among technical replicates, which was found to be in the range of 0.93 to 0.99 (average 0.98). LOWESS algorithm was used for normalisation of intensities [48], and the log_2_ratios were calculated between inhibitor-treated and control groups. Finally, the filtered data were analysed on the basis of the updated genome annotation to eliminate redundancy of the probes per gene [49], [50]. Inspection of box plots revealed that intensities from both dye channels of all technical and biological replicates were in a similar range, showing that no additional normalization steps were necessary.

SAM (Significance Analysis of Microarrays) was used [51] to detect genes with a significant change in transcript level. Data sets for the two treatment periods (24 h and 48 h) were analysed by one-class analysis, in which transcripts were evaluated that showed the same direction of regulation for both time points (sustained regulation direction). Here, genes with a q-value ≤0.01 were considered as significantly differentially transcribed between the inhibitor-treated and the control worm populations comprising protein-coding genes and putative antisense-oriented oligonucleotide probes (labelled as “to be used in analysis  =  YES” in the updated annotation of the array [49], [50]). Spotfire was used for hierarchical clustering [52]. For first functional analyses of differentially transcribed protein-coding genes, Gene Ontology (GO) enrichment analysis was performed [53] using the software tool Ontologizer [54]. Parent-child union [55] was used to detect categories containing enriched genes, and the p-value was adjusted according to Benjamini-Hochberg (BH) correction [56]. For identifying potential candidates for further analyses and their putative involvement in hypothesized pathways, Ingenuity Pathway Analyses (IPA; http://www.ingenuity.com; [57]) were performed in addition, as described before [47]. IPA provides curated information from the literature for human, mouse and rat models about canonical pathways, regulated molecular networks, including signal transduction cascades (of which some are involved in human cancer or other diseases), and regulated transcription factors and their putative targets. To use this tool all *S. mansoni* genes were annotated with the corresponding human homolog and uploaded to IPA along with their corresponding microarray transcription measurements.

The validity of the obtained results of qRT-PCRs (see below) and microarrays was determined by Spearman's rank correlation coefficient (r_s_) [58].

### Quantitative RT-PCR experiments

The reliability of the transcriptional changes detected by microarray analyses was tested by quantitative RT-PCR (qRT-PCR) analyses of a number of genes. RNAs of inhibitor-treated or control couples were isolated by TriFast (PeqLab), and 1 µg each was reverse transcribed (QuantiTect Reverse Transcription kit; Qiagen). Following cDNA dilution 1∶20, qRT-PCRs were done using Rotor Gene Q (Qiagen). Amplification rates were determined by SYBRGreen incorporation (PerfeCTa SYBR Green Super Mix; Quanta). Melting point analyses were done to distinguish between the specific amplification product and unspecific primer-dimer formation following each qRT-PCR analysis. For primer design, the software Primer 3 Plus was used (http://www.bioinformatics.nl/cgi-bin/primer3plus/primer3plus.cgi). The expected amplification products were between 140–160 bp in size. Primers were designed flanking predicted introns to be able to differentiate between cDNA and genomic DNA, and melting points were between 59°C–62°C depending on sequence composition. A list of all primers used, which were commercially synthesised by Biolegio (Netherlands), is shown in Supplementary data S1.

Standard reference genes normally used for relative quantification analyses such as α-tubulin, actin, Cu/Zn SOD (superoxide dismutase), or histone showed regulation following inhibitor treatment. Therefore, absolute quantification was performed on the basis of standard curves generated by purified PCR products (used in dilution series) [59]. Fold changes are given where appropriate. As a basis for comparing microarray and qRT-PCR results, log_2_ratios (treated/control) were calculated as described before [47], [60]. The efficiency of each qRT-PCR was evaluated to be between 90–100%. Spearman's rank correlation coefficient (r_s_) was assessed to validate the ratios obtained from qRT-PCRs and microarrays [58], [61], [62].

### *In silico* analyses

In addition to those already mentioned, the following public domain tools were used: SchistoDB (http://www.schistodb.net; [63]), BLASTx (http://www.ncbi.nlm.nih.gov/BLAST), the Welcome Trust Sanger Institute *S. mansoni* OmniBlast (http://www.sanger.ac.uk/cgi-bin/blast/submitblast/s_mansoni/omni), BLAST (http://blast.ncbi.nlm.nih.gov/), and Gene Cards, which is a database of human genes providing concise genome-related information on all known and predicted human genes, to authenticate IPA-identified gene acronyms (http://www.genecards.org).

## Results

### Homology modeling revealed structural conformity between human and schistosome Abl-kinases

**Figure 1:**
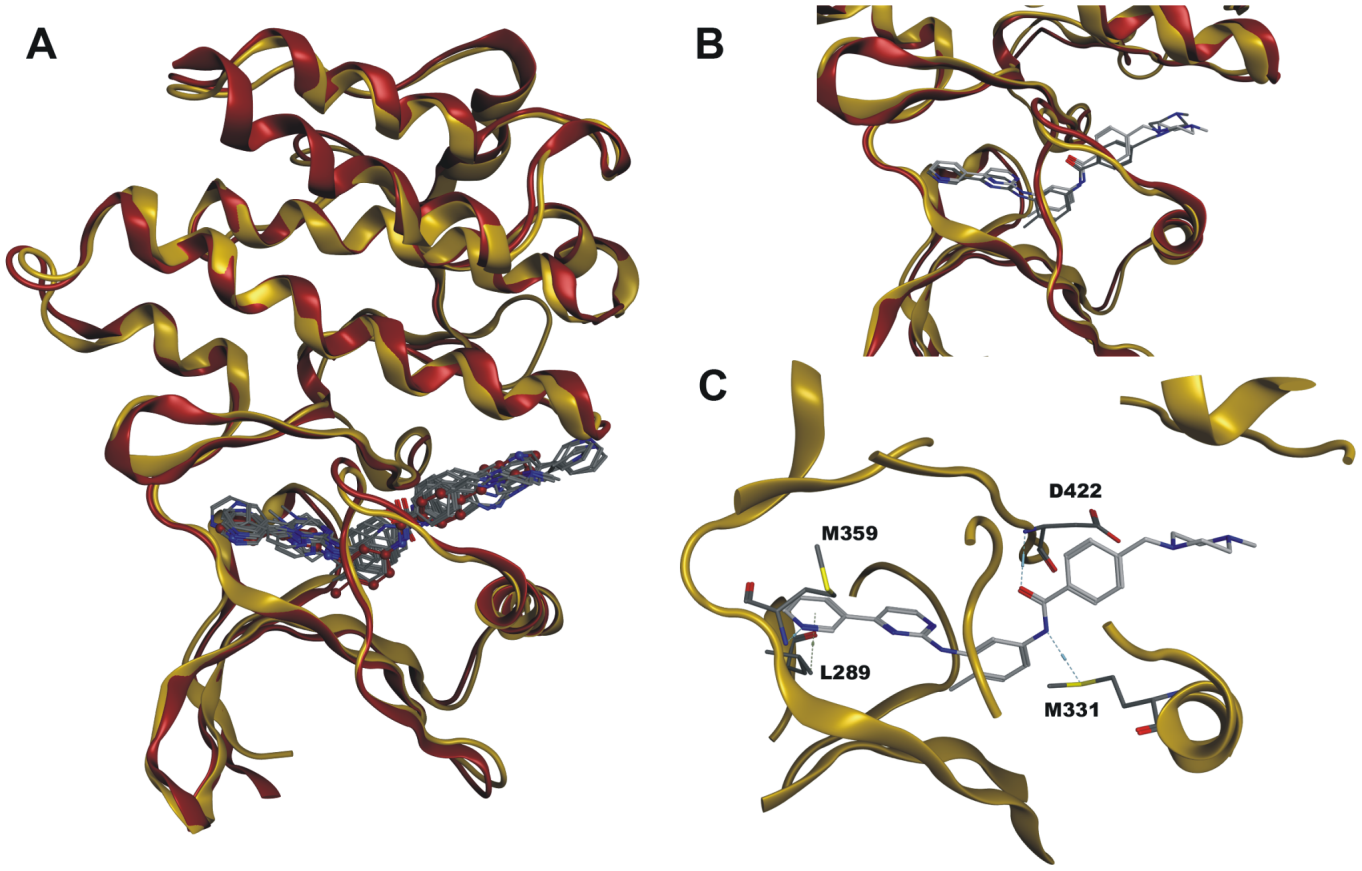
Homology models of human and schistosome Abl kinases. Panels A to C: Structural comparisons of the molecular docking solutions of the SmAbl2 homology model versus the human Abl2 crystal structure (3GVU). Both protein structures are shown in ribbon representation, the SmAbl2 homology model is colored yellow, while the human Abl2 crystal structure is colored red. **A**: Structural superposition of the two protein structures. The ten highest scoring docking solutions for Imatinib in the SmAbl2 homology model were depicted in stick representation in atom color, the crystal structure pose of Imatinib in the human Abl2 crystal structure is depicted in ball-and-stick representation colored red. Hydrogen atoms were omitted for visibility reasons in the depiction. The RMSD value of the template and the model structure resulted in a Cα RMSD (root-mean-square deviation) value of 0.765 Å of the corresponding 245 amino acid residues. **B**: Close-up view of the binding sites of the two protein structures. The top-scored pose of Imatinib in the SmAbl2 homology model was depicted in stick representation with light-grey carbon atoms, the crystal structure pose of Imatinib in human Abl2 was depicted in stick representation with dark grey carbon atoms. **C**: Close-up view of the binding site of SmAbl2 homology model. Imatinib engages in four directed interactions to surrounding amino acid residues, depicted by light blue dashed lines.

**Figure 2:**
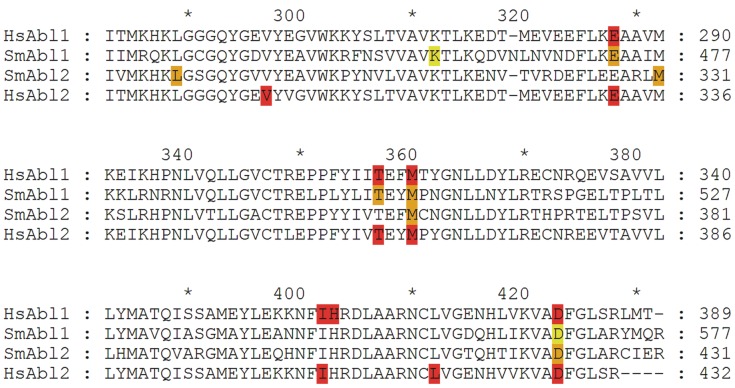
Alignment of the TK domains of human and schistosome Abl kinases. Alignment: Structural alignment of the binding sites of human (HsAbl1, HsAbl2) and *S. mansoni* (SmAbl1, SmAbl2) Abl kinases focused on the highly conserved catalytic tyrosine domain [37]. Amino acid residues partnering in directional interactions with Imatinib are highlighted ocher and red for SmAbl1/2 and HsAbl1/2, respectively. Amino acids D568 and K457 of SmAbl1 are colored light teal, because they are not direct interaction partners to Imatinib but utilize a water molecule.

On the basis of their human counterparts, homology models of the *S. mansoni* Abl kinase 1 and 2 were created which corresponded well with the protein template structures 2HYY (human Abl 1) and 3GVU (human Abl 2). The ten highest scoring docking poses of Imatinib in the homology model of SmAbl2 were found in good structural agreement with the crystal structure pose of Imatinib in the human Abl2 crystal structure (Figure 1 A; SmAbl1 data not shown). The highest scoring docking pose is virtually identical to the crystal structure pose (Figure 1 B). While human Abl2 forms seven directed interactions with Imatinib (Figure 2), for the SmAbl2 homology model four directed interactions were detected (Figure 1 C, Figure 2). Two out of the four SmAbl2 interactions are shared with the human Abl2 interactions (Figure 2). For SmAbl1 the situation was similar. Key residues involved in direct interactions with Imatinib in the human Abl proteins were found to be conserved for all four protein sequences. Four out of the six directed interactions were also detected for the SmAbl1 homology model. In contrast to human Abl 1, one of these residues (D568) did not interact directly with Imatinib; however, it did via an H-bond network involving a water molecule. Since we docked Imatinib to homology models, the recovery of individual directed interactions should not be overstated. Although we docked Imatinib to homology models, the data clearly indicated that Imatinib is able to bind both SmAbl1 and SmAbl2.

### *S. mansoni* Abl-TK activities are inhibited by Imatinib

**Figure 3:**
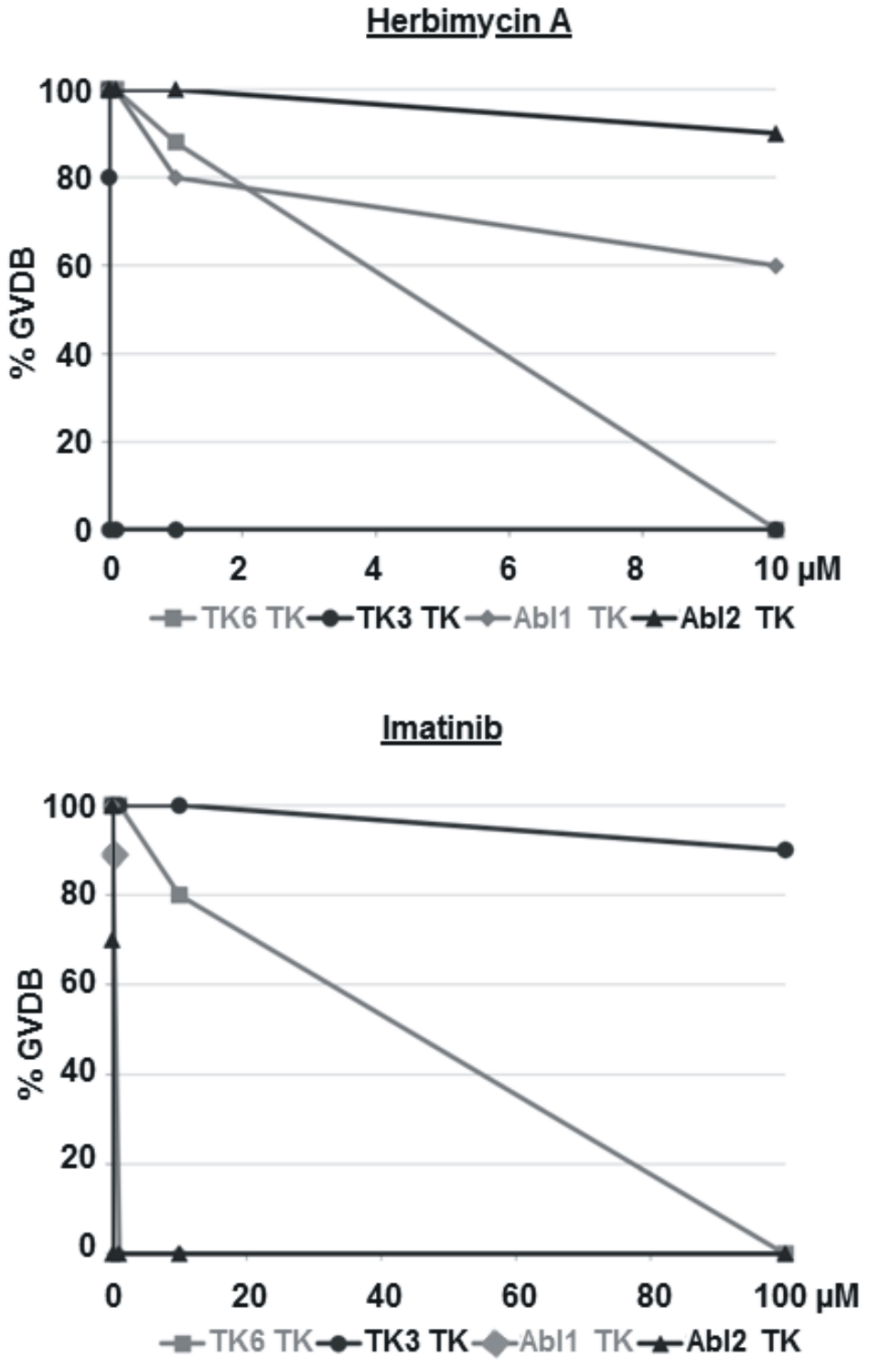
GVBD assays confirming SmAbl2 inhibition by Imatinib. Results of the GVBD assays performed in *Xenopu*s oocytes to analyze schistosome-kinase activities under inhibitor influence. Herbimycin A completely inhibited SmTK3 TK enzymatic activity (circles, black) even at 0.01 µM, whereas 10 µM was needed to stop inhibit SmTK6 (squares, grey)-induced GVBD [38]. At this concentration the GVDB-inducing TK activities of SmAbl1 (rhombus, grey) and SmAbl2 (triangle, black) were reduced by only 40% or 10%, respectively. Imatinib completely inhibited GVBD induced by the TK activities of SmAbl1 (at 1 µM) or SmAbl2 (at 0.1 µM), whereas SmTK6-induced GVBD was stopped at 100 µM, a concentration that reduced SmTK3 TK-induced GVBD by only 10% [38].

An inhibitor swap-like approach [38] was used to test the enzymatic activity of SmAbl2, for its susceptibility towards Imatinib and Herb A. To this end, cRNA encoding the TK domain of SmAbl2 (SmAbl2-TK) was injected into *Xenopus* oocytes under selection conditions using different inhibitor concentrations. GVDB was monitored as read out, and the results compared to SmAbl1, SmTK6 and SmTK3 [38]. Under Herb A selection, SmAbl2-TK induced 100% GVBD at 1 µM and still 90% GVBD at 10 µM (Figure 3). The Src-TK SmTK3 and the Src/Abl-hybrid TK SmTK6 were completely inhibited by Herb A inducing GVBD at concentrations of 0.01 µM (SmTK3), or 10 µM (SmTK6) [39]. Using Imatinib, however, SmAbl2-TK enzymatic activity was reduced to 70% GVBD-inducing capacity at 0.01 µM and completely suppressed GVBD at 0.1 µM. At the latter concentration still 90% GVBD was observed for SmAbl1-TK, whose activity was completely suppressed using 1 µM Imatinib (Figure 3). These results showed that Imatinib effectively inhibits both Abl kinases of *S. mansoni*, which is supported by the modeling data presented above.

### Transcriptome analyses of inhibitor-treated adults exhibited wide-ranging transcriptional changes

Based on our previous findings of remarkable effects of Imatinib on morphology, physiology, and survival of adult *S. mansoni in vitro* [38], [42], we focused on the elucidation of molecular effects induced by this inhibitor. To this end, a large-scale transcriptional analysis was performed using a microarray platform representing nearly the complete *S. mansoni* and *S. japonicum* transcriptomes [46], [47]. Since in previous experiments treatment with 50 µM Imatinib showed slight effects after 24 h and strong effects after 48 h treatment [38], we anticipated that these treatment profiles represented starting (24 h) and peak points (48 h) of the effects induced, thus being interesting for analysis. Therefore, the 50 µM concentration and both time points were chosen for comparative transcriptomics. Based on the results of the GVBD assays it was anticipated that 50 µM Imatinib would not induce potential off-target effects through co-influencing Src kinases such as SmTK3 since its inhibition will not occur using this inhibitor concentration [39; this study]. With this set-up, the expectation was to find genes differing in their transcript levels under inhibitor influence. It was hypothesized that transcript levels of genes strongly regulated by signaling pathways including SmAbl1/2-kinases would show a continuous tendency of regulation during the treatment period of 48 h representing sustained transcriptional changes.

Following microarray hybridization and data evaluation a one-class statistical analysis was performed that revealed sustained transcriptional changes of 1429 significantly differentially transcribed genes which were up-regulated following Imatinib-treatment. Of these, 1094 were protein-coding genes (Supplementary data S2, S3). The remaining transcripts represented antisense RNAs, intronic and UTR sequences. Among the protein-coding genes were candidates coding for serine/threonine PKs, PTKs, proteins with female-preferential or -specific functions such as egg synthesis, transcription factors, muscle-associated proteins, small GTPases, heat-shock proteins, and signal transduction/associated proteins (Supplementary data S4). Furthermore, 939 protein-coding genes were found to be significantly down-regulated. These genes potentially code for cathepsins, lipoproteins, VAL(venome allergen-like) proteins, glutamate receptors and further transporters, the gynecophoral canal protein (GCP), motor and/or muscle proteins, drug efflux proteins, transmembrane receptors, calmodulin and other calcium binding proteins, histones, spermatogenesis-/testis-associated proteins, a morphogen-binding protein, signal transduction (-associated) proteins, and a cell adhesion protein (Supplementary data S5).

GO analyses of differentially transcribed genes revealed ontology categories enriched with genes being up- or down-regulated (BH adjusted p-value ≤0.05; threshold  = 0.1). Examples of GO categories represented in the up-regulated genes were: gene expression and transcription (Biological process), myosin complex (Cellular compenent), kinase activity and transcription factor activity (Molecular Function) (Supplementary data S4, S6). Within the data set of down-regulated genes, GO categories were found for enriched genes coding for functions such as e.g. transmembrane transport or cell surface receptor-linked signalling pathways (Biological process), membrane and microtubule-cytoskeleton/associated complex (Cellular component), and signal transducer activity, transporter activity or cysteine-type endopeptidase activity (Molecular function) (Supplementary data S5, S7).

Using IPA, the following five networks were identified, which were enriched with proteins coded by differentially transcribed genes involved in the following functions: (1) Protein Degradation, Protein Synthesis, Tumor Morphology (*adjusted p-value* 10^−80^), (2) RNA Post-Transcriptional Modification, DNA Replication, Recombination, and Repair, Cellular Assembly and Organization (*adjusted p-value* 10^−69^), (3) Post-Translational Modification, Protein Folding, Carbohydrate Metabolism (*adjusted p-value* 10^−60^), (4) Developmental Disorder, Gene Expression, Genetic Disorder (*adjusted p-value* 10^−40^), and (5) Carbohydrate Metabolism, Drug Metabolism, Lipid Metabolism (*adjusted p-value* 10^−39^) (Supplementary data S8). Among the molecules with the largest fold-changes of transcription was a potential pseudo-glutamine synthetase (LGSN) strongly (about 9-fold) up-regulated, which in the human system is reported to have a chaperone function for the reorganization of intermediate filaments acting as a component of the cytoskeleton [64]. Amongst the strongly down-regulated (about 8 to 11-fold) transcripts were those encoding peptidases such as cathepsins (CatK, CatS, CatL), which are members of the peptidase C1 protein family and known in humans to participate in protein processing during immunological processes and several disease-associated pathologies [65], [66]. In summary, a number of processes were highlighted by both GO analyses and IPA that were influenced by inhibitor treatment pointing to candidate genes such as cathepsins for further analyses.

### Confirmation of selected differentially transcribed genes by quantitative RT-PCR

**Figure 4:**
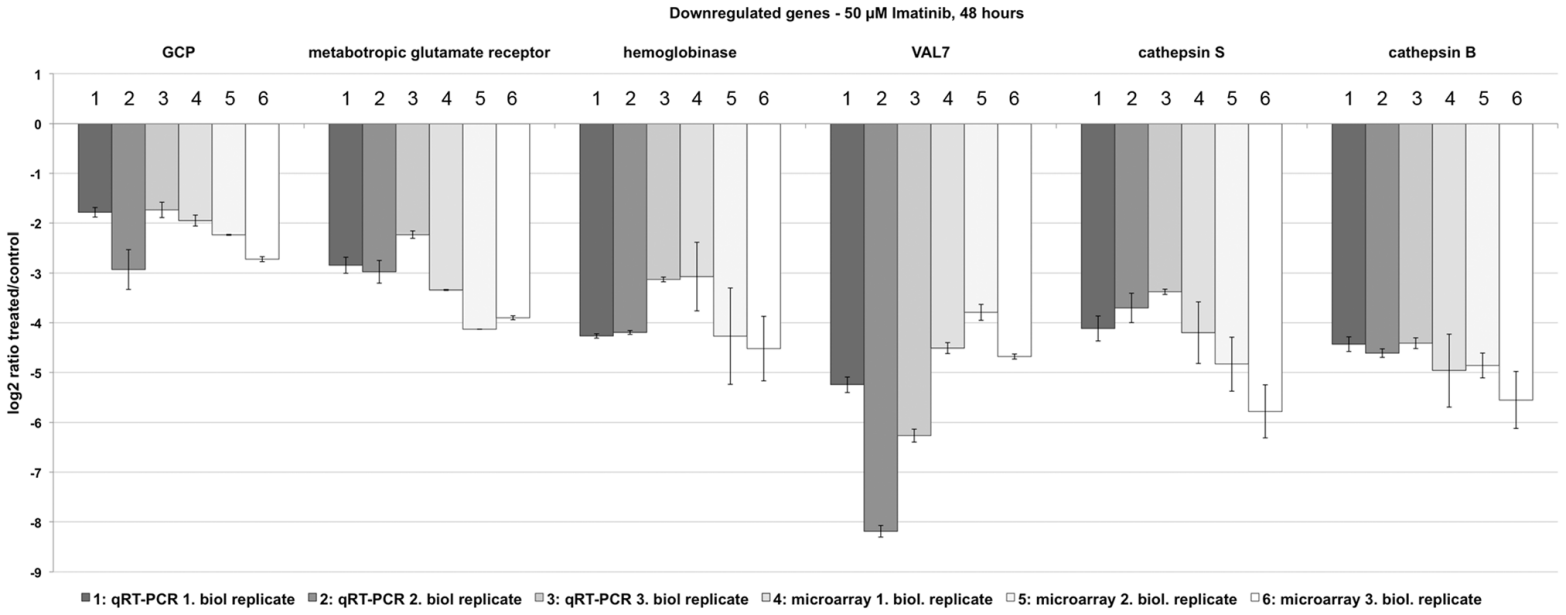
qRT-PCR experiments verifying the down-regulation of genes following Imatinib treatment. Comparative qRT-PCR analyses of genes identified by microarray analyses to be down-regulated in schistosome couples 48 h following Imatinib (50 µM) treatment. The selected genes encode the gynecophoral canal protein GCP (Smp_212710; [72], [82], [83]), the metabotropic glutamate receptor (met. glut. receptor; Smp_128940; [70]), hemoglobinase (Smp_075800), the venom allergen-like protein VAL7 (Smp_070240), cathepsin S (Smp_139240), and cathepsin B (Smp_085180; [67]). Log_2_ratios (treated/control) are given for all biological replicates comparing the results of three independent biological replicates using qRT-PCR (dark gray-shaded columns; numbered 1–3) with the results of three independent microarray analyses (light grey-shaded and white columns; numbered 4–6).

The selection of candidates for qRT-PCR experiments to verify differential transcription was based on GO and IPA results, but also on literature studies including the Imatinib-induced phenotypes in adults obtained previously (negative effects on pairing-stability, oogenesis and spermatogenesis, integrity of the gastrodermis, and locomotion [38], [42]). Since GO and IPA analyses indicated influences of Imatinib treatment on endopeptidase activity and cathepsins, respectively, we chose cathepsin K (Smp_139240) and cathepsin B (Smp_085180). The latter was already shown to be active in the gut [67]. This applies also to the selected hemoglobinase (Smp_075800), which was localized to the gut [68]. Venom allergen-like proteins (VALs) of platyhelminths are members of the SCP/TAPS (Sperm-Coating Protein/Tpx-1/Ag5/PR-1/Sc7) protein superfamily and hypothesized to play not only roles in spermatogenesis but also beyond [69], which led to the choice of VAL7 (Smp_070240). The metabotropic glutamate receptor (Smp_128940; [70]) was selected as a representative for cell surface receptors and its potential role in the nervous system of adult male and female schistosomes [71]. Finally, GCP was included due to its hypothesized role in male-female interaction [72]. The results of the qRT-PCR experiments, which were performed with the RNA of schistosome couples following 48 h Imatinib treatment, confirmed in each case the down-regulation of these transcripts (Figure 4).

**Figure 5:**
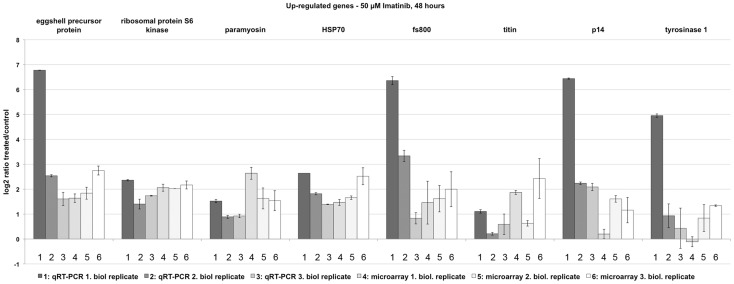
qRT-PCR experiments verifying the up-regulation of genes following Imatinib treatment. Comparative qRT-PCR analyses of genes identified by microarray analyses to be up-regulated in schistosome couples 48 h following Imatinib (50 µM) treatment. The selected genes encode a predicted egg-shell precursor protein (Smp-000430; [47]), a ribosomal protein S6 kinase (rib. protein S6 kinase; Smp_017900), paramyosin (Smp_129550; [73]), HSP70 (Smp_106930; [77], [78]), fs800-like (Smp_000270; [79]), titin (Smp_105020), p14 (Smp_131110; [80]), and the tyrosinase 1 (SmTYR1; Smp_050270; [81]). Log_2_ratios (treated/control) are given for all biological replicates comparing the results of three independent biological replicates using qRT-PCR (dark gray-shaded columns; numbered 1–3) with the results of three independent microarray analyses (light grey-shaded and white columns; numbered 4–6).

Since the GO analysis of up-regulated genes pointed to muscular activities (myosin complex within the ontology cellular component) but also to signal transduction (kinase activity within the ontology molecular function), further candidates were selected for qRT-PCR. Among these were paramyosin (Smp_129550) and titin (Smp_105020), both proteins involved in muscle activity [73], [74], and a ribosomal protein S6 kinase (Smp_017900) due to its potential role as a MAPK-activated PK in signalling processes controlling diverse processes including survival [75], [76]. Furthermore, HSP70 (Smp_106930) was chosen due to its known roles in stress response and signal transduction processes, but also as egg-shell component in schistosomes [77]–[79]. The results of the qRT-PCR analyses confirmed up-regulation in each case (Figure 5).

Unexpectedly, a manual data screen indicated that a number of egg production-related genes were significantly up-regulated following Imatinib treatment such as the egg-shell precursor proteins p14 (Smp_131110; [80]), fs800-like (Smp_000270; [79]), a predicted egg-shell precursor protein (Smp-000430; [47]), and the eggshell protein cross-linking tyrosinase SmTYR1 (Smp_050270; [81]). Also for these genes, qRT-PCR confirmed up-regulation following Imatinib treatment (Figure 5).

The results obtained for the qRT-PCR analyses of all studied genes significantly correlated to the microarray data according to Spearman's Correlation Coefficient (r_s_ = 0.784, p<0.001; [58]).

Extended analyses indicated that Imatinib treatment can lead to a sustained effect on specific genes. By qRT-PCR analyses of RNA of couples treated with Imatinib for 24 h or 48 h, the amounts of gene transcripts increased or decreased over time as demonstrated exemplary by the analyses of three genes. Compared to 24 h treatment higher transcript levels were determined for the ribosomal S6 kinase after 48 h (Supplementary data S9), whereas transcript levels declined for hemoglobinase and GCP from 24 h to 48 h (Supplementary data S10, S11).

### Merging microarray data sets of Imatinib and TRIKI treatments of adult schistosomes provided first evidence for a TGFβ-pathway contribution

Since the couples used for these analyses were separated before freezing, we checked whether pairing had an influence on the transcription of the GCP gene, which was hypothesized before to be a target of a TGFβ-pathway but also a male factor contributing to pairing-dependent female maturation [72], [82], [83]. A qRT-PCR analysis using actin as reference gene showed that the status of pairing had no significant influence on the GCP transcript level, since there was no significant difference in transcript levels comparing males with and without pairing experience or males separated from their female partners (Supplementary data S12). This finding is also supported by results of a recent study comparing the transcriptomes of pairing-experienced males versus naïve males using microarrays, SuperSAGE and also qRT-PCR, in which no evidence for an influence of pairing on GCP expression was found [84]. Thus the decrease of the GCP transcript level following Imatinib treatment represented an inhibitor-specific effect.

**Figure 6:**
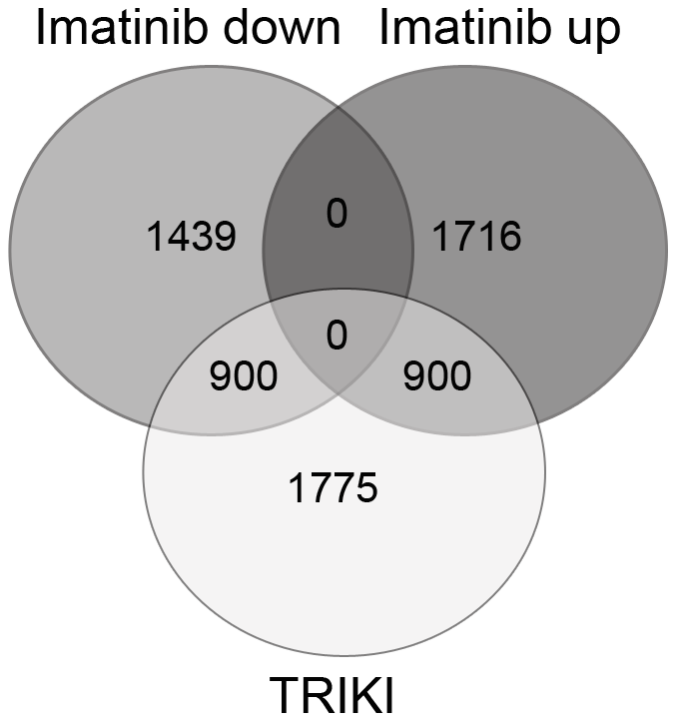
Venn diagram of genes down- or up-regulated following Imatinib or TRIKI treatment. Merging analyses represented by a Venn diagram of the intersections of significant differentially transcribed down- or up-regulated genes following Imatinib treatment with those differentially (up- and down-) regulated upon TRIKI treatment [47]. The direction of regulation following Imatinib treatment (down/up) is shown within the header. Given numbers correlate with the numbers of significant differentially transcribed genes for these data sets (see text). The overlapping regions of the circles contain the corresponding differentially transcribed genes in these data sets.

Recent years have provided compelling evidence for a prominent role of TGFβ signalling in schistosome biology [27], [47], [84], [85], [86]. Results of a previous study suggested GCP to be part of TGFβ signalling pathways [82]. Furthermore, the effect of a specific TβRI kinase-inhibitor (TRIKI) was investigated in schistosomes. *In vitro*-culture experiments with couples provided first evidence for a role of the TGFβ pathway during the regulation of mitotic activity and egg production [26]. Subsequently, it was shown by microarray analysis using the same technical platform that genes contributing to these processes, such as egg shell-forming genes, were slightly up-regulated upon TRIKI treatment [47]. Analysing the microarray data following Imatinib treatment in the present study we observed that a number of specific genes were differentially regulated that had shown up in the previous analysis as well. Since there is evidence from the literature that Abl-kinases can be part of TGFβ signalling pathways [87], [88] we investigated whether this may apply for schistosomes as well and compared both data sets in a merging analysis. To this end the Imatinib data set of this study and the TRIKI data set of the previous microarray study [47] were compared using a significance q-value of ≤0.05 to identify a comprehensive set of differentially transcribed genes found in common in the two conditions. This approach identified 6754 differentially transcribed protein-coding genes in total, of which 1800 were common in both data sets. The merging analyses finally indicated that out of 2339 genes found in this study to be down-regulated upon Imatinib treatment, 900 matched those differentially (up- and down-) regulated upon TRIKI treatment. Out of these 480 were up-regulated and 420 down-regulated by TRIKI. Furthermore, out of 2616 genes found in this study to be up-regulated upon Imatinib treatment, 900 corresponded to those differentially regulated by TRIKI. Out of these 822 were up-regulated and 78 down-regulated by TRIKI. By definition, no gene was found within the intersection of Imatinib up- and down-regulated genes (Figure 6; Supplementary data S13, S14).

## Discussion

Based on our previous results on the physiology and morphology of adult schistosomes treated by Imatinib [38], our present study aimed at identifying transcriptional processes influenced by this inhibitor. To confirm that Imatinib targets not only the Abl kinase SmAbl1 as shown by biochemical analyses before [39], we investigated its inhibitor effect on SmAbl2. SmAbl1 and SmAbl2 are the only true Abl-kinases present within the genome of *S. mansoni* [50] in contrast to SmTK6 which represents a Src/Abl hybrid kinase being less susceptible to Imatinib [39; this study]. By competitive GVBD assays in *Xenopus* oocytes expressing these kinases we determined specific effects of Imatinib on both Abl1 and Abl2 kinases. Although their susceptibilities differed by a factor of 10, the results obtained in this and the previous study [39] clearly confirmed their target roles, but also that Src-like and true Src kinases such as SmTK3 are not affected at the Abl-effective concentrations of Imatinib, which reduced the probability of off-target effects. These experimental data were well supported by the modeling and docking data generated confirming that Imatinib is able to bind to both schistosome Abl kinases.

With regard to results of the *in vitro* study showing increasing physiological and morphological effects between 24 h and 48 h treatment using 50 µM Imatinib [38], we performed transcriptional profiling for these time-points to get a broader view of molecular processes potentially affected by Imatinib. Microarray and subsequent bioinformatics analyses revealed a broad spectrum of genes being differentially regulated following inhibitor treatment. The transcription of genes involved in male-female interaction, gut physiology, muscle activities, and egg production were among those highlighted by the analyses. For qRT-PCR verification a number of genes were selected with regard to GO and IPA results but also to the phenotypes obtained in the preceding *in vitro* study, which comprised reduced pairing stability (i), reduced sizes of the gonads of both genders combined with disturbed spermatocyte/oocyte differentiation (ii), a degradation of the gastrodermis (iii), and tremor-like movements pointing to altered locomotion activity (iv) [38]. In each case qRT-PCR and microarray results correspondingly showed sustained transcriptional changes and reduced transcript levels in each case for (i) GCP, a fascicle I-like cell adhesion molecule hypothesized to be involved in male-female interaction [72], [83], (ii) VAL-7, a member of the sperm-coating protein SCP/TAPS superfamily [89], [90] that was found in *S. mansoni* to be expressed in the esophageal gland in larvae, and adult males and females [91], (iii) a hemoglobinase and the cathepsins B and S, of which hemoglobinase and cathepsin B were already shown to be active in the gut [67], [92]–[94], (iv) as well as a metabotropic glutamate receptor. Interestingly, previous studies indicated that Abl kinases regulated lysosome functions, especially autophagy by organizing the localization and activity of lysosomes, glycosidases and cathepsins, suggesting that Abl is involved in processes regulating digestion and removal of self- and foreign material [95], [96]. Metabotropic glutamate receptors have been discussed in the context of seizure-like behavior, defined as paroxysms resulting in disruption of normal locomotor-system activity in planaria [97]. Whether this tremor-like phenotype in schistosomes (iv) is also accompanied by higher transcript levels detected for the muscle protein genes paramyosin and titin, of which the latter determines muscle elasticity, stability, and contraction velocity [98], remains unclear at this stage. In contrast, the up-regulation of the stress protein gene HSP70 following inhibitor treatment meets the expectations as well as higher transcript levels of the ribosomal S6 kinase, a signaling molecule involved in cell growth, proliferation, but also survival [99]. Thus the differential regulation of these genes corresponded well to the phenotypes observed *in vitro*. Furthermore, a comparison of both time-points used for the analysis (24 h/48 h) showed strong and sustained transcriptional changes by microarrays but also on the basis of qRT-PCRs of selected candidate genes, which indicated an enduring influence of the Abl kinase pathway on these genes.

Compared to transcriptome studies in *S. mansoni* or *S. japonicum* after exposure to PZQ *in vitro* [100], [101] or *in vivo* [102], a number of differences can be noted that indicate dissimilar processes affected by this established drug and Imatinib. Using a recent *in vivo* model of *S. japonicum* PZQ led to an up-regulation among others of genes associated with muscle function, lipid and ion regulation, and drug resistance in treated males [102]. In females, fewer genes seemed to be affected (up-regulated), examples are involved in pathogen defense, general detoxification, drug resistance and calcium regulation [102]. Similar findings were made in *in vitro* studies with adult *S. mansoni* showing that genes encoding multiple drug transporter as well as calcium regulation, stress and apoptosis-related proteins were up-regulated [101]. In contrast to these findings we observed a down-regulation of genes coding for lipoproteins, motor and/or muscle proteins, drug efflux proteins, calmodulin and other calcium binding proteins in *S. mansoni* couples following Imatinib treatment. With respect to apoptosis-related genes the picture is puzzling since there much variation within all PZQ and Imatinib data sets and little correspondence among these different data. This justifies no precise conclusion on the participation of defined apoptosis-related signaling processes in the primary effects on schistosomes caused by these drugs.

Surprisingly, we also identified genes to be up-regulated that contribute to egg formation such as p14, fs800-like, a predicted egg-shell precursor protein gene, and tyrosinase 1, a gene involved in final egg-shell synthesis [79], [81]. This was unexpected since we observed reduced egg production in Imatinib-treated schistosome couples. However, egg production is a complex process and may be influenced by further genes of which some, yet unknown to be important for this process, may be down-regulated by Imatinib, while the known egg-formation genes might be up-regulated to compensate for the overall reduced egg output in Imatinib-treated schistosomes.

Conspicuously, the higher transcript levels of these egg formation-related genes resembled the results obtained in a recent microarray study where the effect of TRIKI, a TGFβRI-kinase inhibitor, was investigated on transcriptional profiles in adult schistosome couples *in vitro*. TRIKI led to an increase of transcript levels of the same egg formation-related genes in paired females in contrast to Herb A, a Src kinase inhibitor, which reduced the transcript levels of these genes. From this it was concluded that a TGFβ and a Src kinase pathway cooperatively control egg formation processes in a balanced manner in schistosomes assigning repressing (TGFβ/TGFβRI-pathway) and inducing (Src-pathway) tasks [47]. This and the finding of GCP and egg formation-associated genes as common target molecules of TGFβ [82] as well as SmAbl1/2-influenced molecular processes [this study], prompted us to evaluate the TRIKI-related against the Imatinib-related microarray data sets. Comparing the total amounts of differentially transcribed genes about 27% (1800 out of 6754) were present in both data sets, of which about 50% (900 or 900 out of 1800) were differentially regulated and about 70% (420 and 822 out of 1800) in the same direction. Thus many genes significantly differentially transcribed upon TRIKI- and Imatinib treatment overlapped. This clearly indicates a potential association of TGFβRI-mediated and Abl kinase-containing pathways in schistosomes. Beyond the fact that egg formation-associated genes such as fs800-like, p14, egg shell precursor, and tyrosinase 1 [47; this study] as well as GCP became noticeable as common targets, SmAbl1/2 transcripts and TGFβRI-transcripts were found in the same tissues by *in situ* hybridization, mainly in the gonads [38], [103]. In conclusion, it appears very likely that the schistosome Abl kinase(s) are among other possibilities members of signalling pathways induced by TGFβ. Such a molecular connection has been shown before, demonstrating c-Abl as a Smad-independent component of TGFβ signaling pathways and mediator of TGFβ-driven proliferation in human fibroblasts [87], [104], [105].

Our previous *in vitro* studies exhibited strong effects of Imatinib on schistosome morphology, physiology and survival *in vitro* suggesting that this compound may be one of the candidates for the design of alternative strategies to fight schistosomiasis [38], [42], [43]. This was confirmed by an independent approach recently, which reproduced similar phenotypes *in vitro*, although a first *in vivo* experiment failed [106]. Nonetheless, the data obtained in this study support the conclusion that Imatinib exerts broad negative effects on worm physiology substantiating the hypothesized role of PKs as potential targets [25], [28], [36], [42], [43]. In this respect it was encouraging to note that according to our microarray analysis also multidrug resistance (MDR) genes (Smp_089200, Smp_151290) were among the significantly down-regulated genes following Imatinib treatment. Thus they may represent additional targets of Abl-kinase-containing pathways. Future studies could also aim at analyzing the molecular networks controlling the expression of such MDR genes and their substrate specificities in more detail. Depending on the substrates transported by these MDRs, and with respect to treatment strategy and efficacy, the suppression of MDR genes as an additional consequence of inhibitor application would represent a potential side effect that is most welcome.

## Supporting Information

Data S1 List of primers used for qRT-PCRs. Smp numbers of the target genes, primer sequences (f  =  forward, r  =  reverse), and annealing temperatures (Tm) used are given.
(DOCX)

Data S2 Hierarchical clustering of differentially transcribed genes (q = 0.1%) following Imatinib treatment. Summarized are three biological replicates for each time point analysed (24 h, 1–3; 48 h, 1–3). Genes with repressed transcription (down-regulated) are colored in green, and genes with enhanced transcription (up-regulated) in red.
(TIF)

Data S3 List of all significantly differentially transcribed genes following Imatinib treatments (24 h and 48 h; q = 0.1%) according to the one-class analysis. This list is subdivided in up- and down-regulated genes.
(XLSX)

Data S4 List of selected genes up-regulated after Imatinib treatment (q = 0.1%). Besides the Gene ID number, relative transcript ratios are given for both time-points (24 h and 48 h) as well as annotations and functional categories.
(DOCX)

Data S5 List of selected genes down-regulated after Imatinib treatment (q = 0.1%). Besides the Gene ID number, relative transcript ratios are given for both time-points (24 h and 48 h) as well as annotations and functional categories.
(DOCX)

Data S6 Gene Ontology (GO) categories are listed enriched with significantly differentially transcribed genes (BH adjusted p-value ≤0.05; threshold  = 0.1) up-regulated following Imatinib treatment.
(XLSX)

Data S7 Gene Ontology (GO) categories are listed enriched with significantly differentially transcribed genes (BH adjusted p-value ≤0.05; threshold  = 0.1) down-regulated following Imatinib treatment.
(XLSX)

Data S8 Summary of the IPA analysis [57] containing lists of networks, biological functions, canonical pathways, and the top molecules being down- and up-regulated based on the relative fold changes of expression.
(XLSX)

Data S9 Summary of the qRT-PCR and microarray analyses which show the sustained effect of Imatinib treatment (24 h or 48 h) on the transcript level of the ribosomal S6-kinase gene. Log_2_ratios (treated/control) are comparing the results of three independent biological replicates using qRT-PCR (24 h and 48 h, columns 1–3, light gray) with the results of three independent microarray analyses (24 h and 48 h, columns 4–6, dark gray).
(TIF)

Data S10 Summary of the qRT-PCR and microarray analyses which show the sustained effect of Imatinib treatment (24 h or 48 h) on the transcript level of the hemoglobinase gene. Log_2_ratios (treated/control) are given comparing the results of three independent biological replicates using qRT-PCR (24 h and 48 h, columns 1–3, light gray) with the results of three independent microarray analyses (24 h and 48 h, columns 4–6, dark gray).
(TIF)

Data S11 Summary of the qRT-PCR and microarray analyses which show the sustained effect of Imatinib treatment (24 h or 48 h) on the transcript level of the GCP gene. Log_2_ratios (treated/control) are given comparing the results of three independent biological replicates using qRT-PCR (24 h and 48 h, columns 1–3, light gray) with the results of three independent microarray analyses (24 h and 48 h, columns 4–6, dark gray).
(TIF)

Data S12 Result of the transcript level of GCP determined by qRT-PCR with RNA from males cultured *in vitro*, which either have never been paired with a female (1), or were separated from a female for five days (2), or were kept in culture paired with females (3) before they were separated to perform the analysis (n = 3). Actin transcript levels were determined as reference in each case.
(TIF)

Data S13 List of genes identified in the merging analysis to be down-regulated after Imatinib treatment and differentially regulated (up or down) following TRIKI treatment. Besides the Gene ID number, relative transcript ratios are given for both time-points (24 h and 48 h) as well as annotations and functional categories.
(XLSX)

Data S14 List of genes identified in the merging analysis to be up-regulated after Imatinib treatment and differentially regulated (up or down) following TRIKI treatment. Besides the Gene ID number, relative transcript ratios are given for both time-points (24 h and 48 h) as well as annotations and functional categories.
(XLSX)
