# Supplementary material for: Imatinib Treatment Causes Substantial Transcriptional Changes in Adult Schistosoma mansoni In Vitro Exhibiting Pleiotropic Effects
Source: PLoS Negl Trop Dis. 2014 Jun 12;8(6):e2923. doi: 10.1371/journal.pntd.0002923 (PMC4055459; doi:10.1371/journal.pntd.0002923)
Supplement: Data S1 — List of primers used for qRT-PCRs. Smp numbers of the target genes, primer sequences (f = forward, r = reverse), and annealing temperatures (Tm) used are given. (DOCX) [file pntd.0002923.s001.docx]

**Supplementary data S1:**

**Primers used for the cloning of tyrosine kinases and for qRT-PCR**

Sequences of the tyrosine kinase (TK) domains of SmAbl1, SmAbl2, SmTK6, and SmTK3 were obtained by PCR amplification using the respective full-length kinase sequences as templates and the following 5’ and 3’ primers:

| **Gene** | **Primer** | **Primer sequence (5’** - **3’)** |  |
| --- | --- | --- | --- |
| SmAbl1-TK domain | SmAbl1-TK-pcDNA3B-5’ | TGGAATTCTATGCCCGAAATTATAATGCGTC |  |
|  | SmAbl1-TK-pcDNA3B-3’ | GCGGCCGCCTTGTTCCAG-TTCCGCA |  |
| SmAbl2-TK domain | SmAbl2-TK-pcDNA3B-5’  SmAbl2-TK-pcDNA3B-3’ | GGATCCATGTCAGGACAGTATGGTGTAGTATATGAG  GCGGCCGCCTTCCAATTGATTCAATATATCAGA |  |
| SmTK6-TK domain | SmTK6-TK-pcDNA3B-5‘  SmTK6-TK-pcDNA3B-3‘ | GGATCCATGTTTGCAATTATCCGT GATAG  GCGGCCGCCTAAATATTGAGCTTCTGTGTGCG |  |
| SmTK3-TK domain | SmTK3-TK-pcDNA3B-5‘  SmTK3-TK-pcDNA3B-3‘ | GGATCCATGCTCATTGATAAATGGGAAATTCC  GCGGCCGCCTGGTTGCTCATCTTC ACAGA |  |
|  |  |  |  |

These 5’ and 3’ primers contained *Bam*HI and *Not*I sites, respectively, to control the direction of the insertion of amplification products into the plasmid pcDNA3.1B (Invitrogen), which contained a T7 promoter for *in vitro* transcription.

**Primers used for qRT-PCRs**

| **Gene** | **Primer pair** | **Primer sequence (5’** - **3’)** | **Tm [°C]** |
| --- | --- | --- | --- |
| eggshell precursor protein | Smp_000430-f | CCGTAAAGGTGGTGGC | 62 |
|  | Smp_000430-r | TTGAATGTTGAATAGCCTTGC | 62 |
| ribosomal protein S6 kinase | Smp_017900-f2 | ACCACTTGTTTGAGTTCACC | 59 |
|  | Smp_017900-r2 | GACAAGCCAGCTAAGCG | 60 |
| paramyosin | Smp_129550-f | ACAGAACTTGCGAAACAGC | 61 |
|  | Smp_129550-r | CGGATACACCAGCTTCG | 60 |
| Hsp70 | Smp_106930-f | GCGTGCACTGACTAAGGAC | 60 |
|  | Smp_106930-r | CTGCTTCCCAGTACCCTTG | 60 |
| fs800 | Smp_000270-f | CAGCCGAAAAAGTCAAACA | 61 |
|  | Smp_000270-r | CCCTTTTGCATCGTAAGCT | 62 |
| titin | Smp_105020-f2 | CTAGAGGAGCCTGGCG | 60 |
|  | Smp_105020-r2 | CATTTCCACAGTCAACAGG | 59 |
| p14 | Smp_131110-f | CCTATGGCGGTGATTATGG | 60 |
|  | Smp_131110-r | GGCTGGGTTTGTAAGTGC | 60 |
| SmTYR1 | Smp_052070-f | CAGGGACAGCAAGCAATG | 60 |
|  | Smp_052070-r | CTTGAATGTCCAGGACGAA | 60 |
| GCP | Smp_212710-f2 | AATGTTCCACCCGACTTC | 60 |
|  | Smp_212710-r2 | CCAACCATTATCCGTGG | 60 |
| metabotropic glutamate receptor | Smp_128940-f2 | AGTCGAATGTACGGAGTGG | 60 |
|  | Smp_128940-r2 | GATGCTGGACCACCTAGAG | 60 |
| hemoglobinase | Smp_075800-f | AACGAACACTCGATCAACAG | 60 |
|  | Smp_075800-r | TTGGAGGTTCATCGTTCTC | 60 |
| VAL7 | Smp_070240-f | GGTCAGAGTATCGCTTTCG | 60 |
|  | Smp_070240-r | GGTATCGGCGAATGCTA | 61 |
| cathepsin S | Smp_139240-f | CCAGAATACGTTGATTGGAGA | 62 |
|  | Smp_139240-r | TGGAGTAAGAGTTCCTGTCTTGA | 62 |
| cathepsin B | Smp_085180-f2 | AACCGTTATTTGAAAATTGATCA | 61 |
|  | Smp_085180-r2 | TGAATTCGTTTCCCACCT | 61 |
| SmTK4 | Smp_0149460-f | GGTGGAGGAAATTTTGGTC | 61 |
|  | Smp_0149460-r | TCAATTGAGCCATTGTGC | 61 |
| sperm flagellar- like protein | Smp_060160-f | GGTGTTCTGATGGCTGAAG | 61 |
|  | Smp_060160-r | CGTCATCCGAGAGCTCA | 61 |
| bruno-like RNA binding protein | Smp_041280-f | CGGCAAATCTCAATGGTC | 62 |
|  | Smp_041280-r | GGCCACAATCCATAAGTTG | 60 |
| actin | Smp_161930-f  Smp_161930-r | GGAAGTTCAAGCCCTTGTTG  TCATCACCGACGTAGCTGTC | 60  60 |
