# Supplementary figures and images for: Imatinib Treatment Causes Substantial Transcriptional Changes in Adult Schistosoma mansoni In Vitro Exhibiting Pleiotropic Effects
Source: PLoS Negl Trop Dis. 2014 Jun 12;8(6):e2923. doi: 10.1371/journal.pntd.0002923 (PMC4055459; doi:10.1371/journal.pntd.0002923)

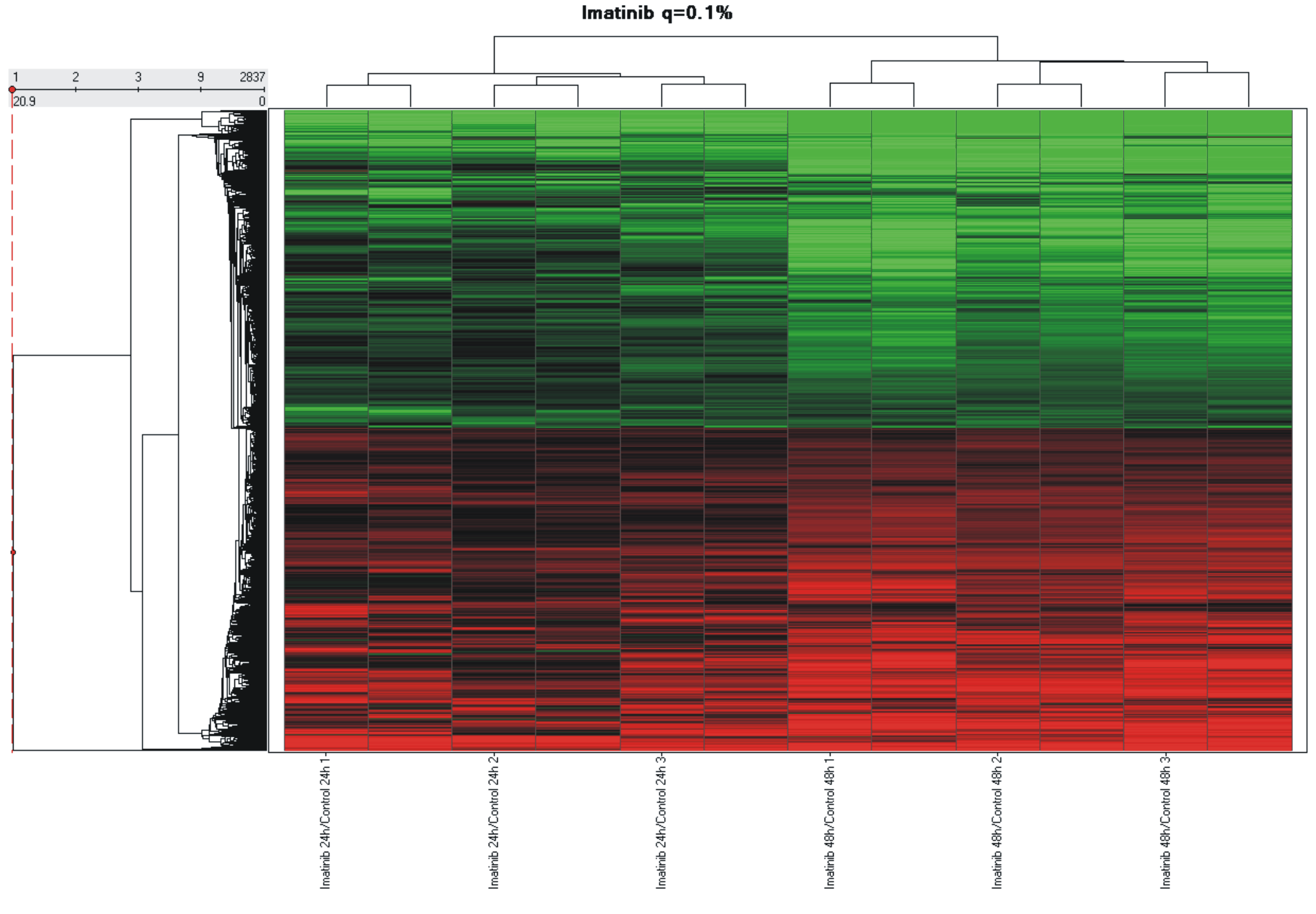

Supplement: Data S2 — Hierarchical clustering of differentially transcribed genes (q = 0.1%) following Imatinib treatment. Summarized are three biological replicates for each time point analysed (24 h, 1–3; 48 h, 1–3). Genes with repressed transcription (down-regulated) are colored in green, and genes with enhanced transcription (up-regulated) in red. (TIF) [file pntd.0002923.s002.tif]

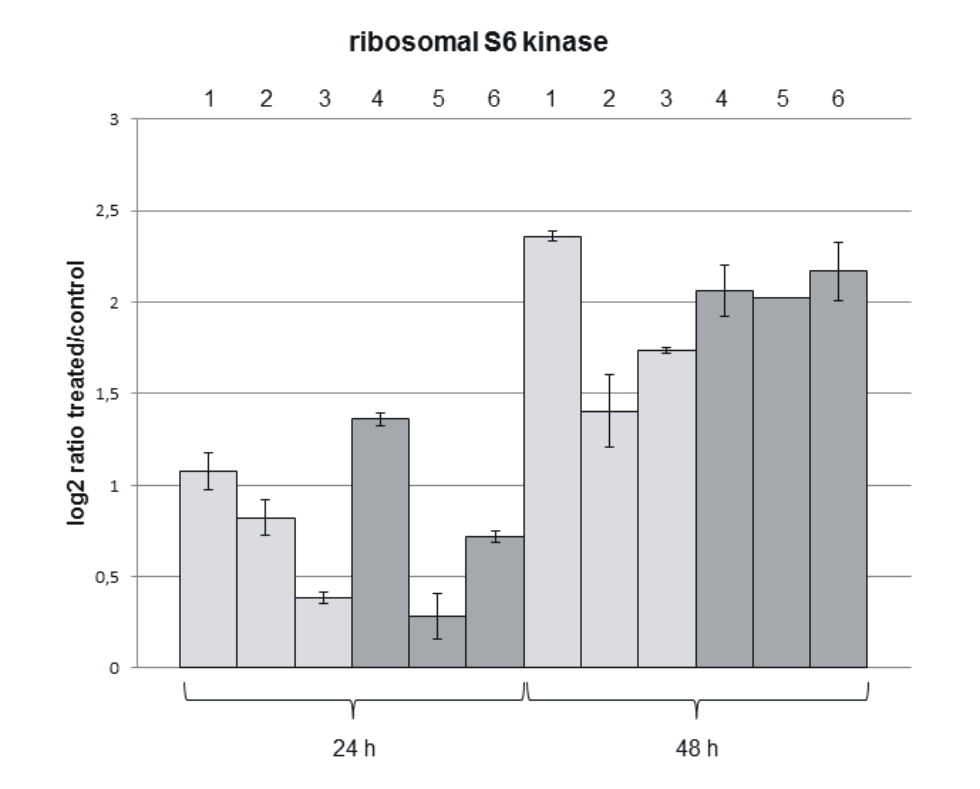

Supplement: Data S9 — Summary of the qRT-PCR and microarray analyses which show the sustained effect of Imatinib treatment (24 h or 48 h) on the transcript level of the ribosomal S6-kinase gene. Log2ratios (treated/control) are comparing the results of three independent biological replicates using qRT-PCR (24 h and 48 h, columns 1–3, light gray) with the results of three independent microarray analyses (24 h and 48 h, columns 4–6, dark gray). (TIF) [file pntd.0002923.s009.tif]

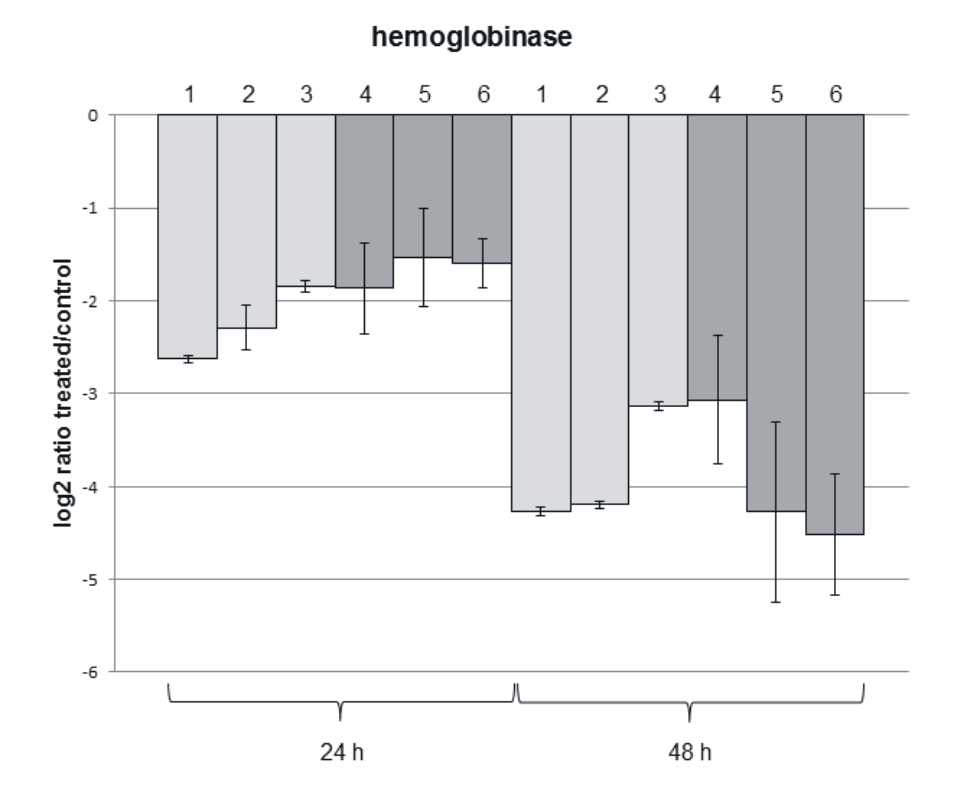

Supplement: Data S10 — Summary of the qRT-PCR and microarray analyses which show the sustained effect of Imatinib treatment (24 h or 48 h) on the transcript level of the hemoglobinase gene. Log2ratios (treated/control) are given comparing the results of three independent biological replicates using qRT-PCR (24 h and 48 h, columns 1–3, light gray) with the results of three independent microarray analyses (24 h and 48 h, columns 4–6, dark gray). (TIF) [file pntd.0002923.s010.tif]

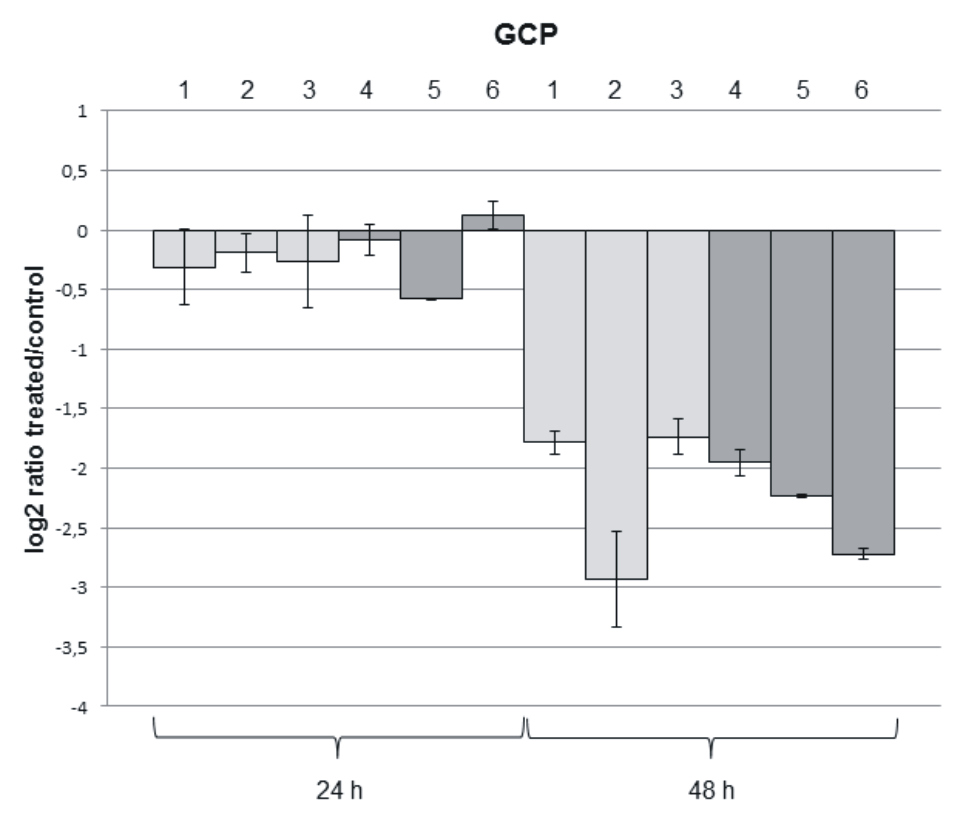

Supplement: Data S11 — Summary of the qRT-PCR and microarray analyses which show the sustained effect of Imatinib treatment (24 h or 48 h) on the transcript level of the GCP gene. Log2ratios (treated/control) are given comparing the results of three independent biological replicates using qRT-PCR (24 h and 48 h, columns 1–3, light gray) with the results of three independent microarray analyses (24 h and 48 h, columns 4–6, dark gray). (TIF) [file pntd.0002923.s011.tif]

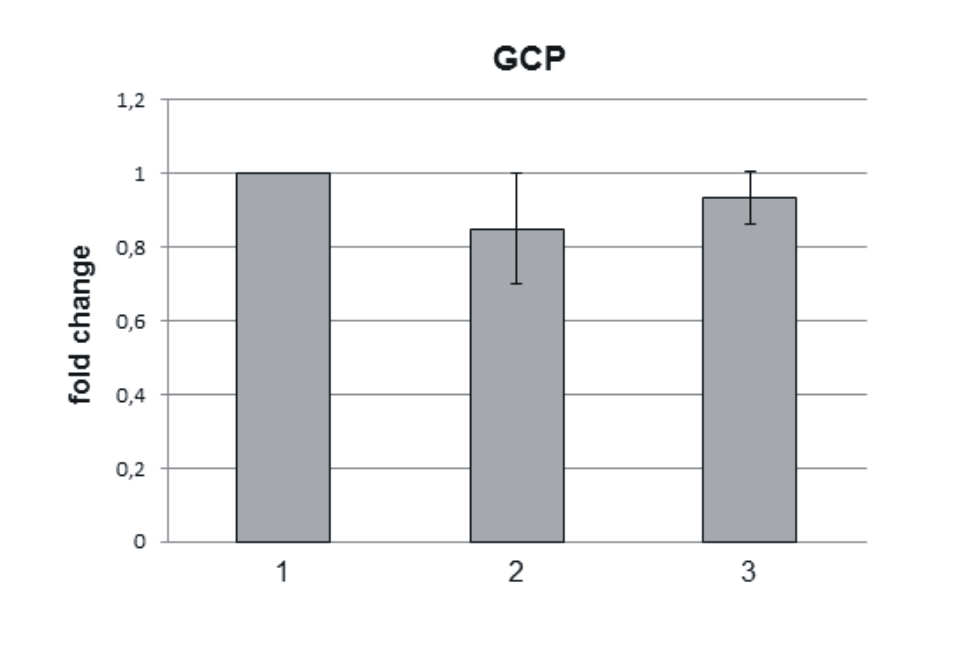

Supplement: Data S12 — Result of the transcript level of GCP determined by qRT-PCR with RNA from males cultured in vitro, which either have never been paired with a female (1), or were separated from a female for five days (2), or were kept in culture paired with females (3) before they were separated to perform the analysis (n = 3). Actin transcript levels were determined as reference in each case. (TIF) [file pntd.0002923.s012.tif]
