# Supplementary material for: Imatinib Treatment Causes Substantial Transcriptional Changes in Adult Schistosoma mansoni In Vitro Exhibiting Pleiotropic Effects
Source: PLoS Negl Trop Dis. 2014 Jun 12;8(6):e2923. doi: 10.1371/journal.pntd.0002923 (PMC4055459; doi:10.1371/journal.pntd.0002923)
Supplement: Data S4 — List of selected genes up-regulated after Imatinib treatment (q = 0.1%). Besides the Gene ID number, relative transcript ratios are given for both time-points (24 h and 48 h) as well as annotations and functional categories. (DOCX) [file pntd.0002923.s004.docx]

Supplementary data 4

| **Gene ID** | **Ratio 24h** | **Ratio 48h** | **Annotation** | **Functional category** |
| --- | --- | --- | --- | --- |
|  | | | | |
| Q2_P20055 | 2,14 | 2,74 | SNF1-like kinase | serine/threonine protein kinases |
| Q2_P16868 | 0,94 | 2,24 | protein kinase C |  |
| Q2_P20645 | 0,79 | 2,08 | ribosomal protein S6 kinase 2 alpha |  |
| Q2_P07423 | 0,47 | 1,30 | ribosomal protein S6 kinase |  |
| Q2_P31690 | 0,69 | 2,08 | mitogen-activated protein kinase |  |
| Q2_P32817 | 1,02 | 1,58 | protein serine/threonine kinase |  |
| Q2_P01085 | 0,98 | 1,48 | G protein-coupled receptor kinase |  |
| Q2_P12669 | 0,83 | 1,60 | dual-specificity tyrosine-(Y)-phosphorylation regulated kinase |  |
| Q2_P19168 | 0,76 | 1,59 | mitogen-activated protein kinase kinase kinase |  |
| Q2_P05720 | 0,61 | 0,98 | mitogen-activated protein kinase-activated protein kinase |  |
| Q2_P21143 | 0,78 | 1,56 | serine/threonine protein kinase |  |
| Q2_P05160 | 1,01 | 1,16 | serine/threonine protein kinase |  |
| Q2_P01089 | 0,83 | 1,32 | serine/threonine protein kinase |  |
| Q2_P34148 | 0,48 | 1,40 | serine/threonine protein kinase |  |
| Q2_P16563 | 0,49 | 1,32 | serine/threonine protein kinase |  |
| Q2_P20754 | 0,75 | 1,02 | rio1 (rio kinase 1) |  |
| Q2_P23316 | 0,37 | 0,93 | rio2 (rio kinase 2) |  |
| Q2_P06077 | 0,46 | 1,21 | serine/threonine protein kinase |  |
| Q2_P35511 | 0,69 | 0,64 | serine/threonine protein kinase |  |
| Q2_P07483 | 0,25 | 1,03 | cyclin-dependent kinase |  |
| Q2_P29491 | 0,31 | 0,94 | serine/threonine protein kinase |  |
| Q2_P26498 | 0,46 | 0,71 | serine/threonine protein kinase |  |
| Q2_P05902 | 0,21 | 0,95 | serine/threonine protein kinase |  |
|  | | | | |
| Q2_P19247 | 1,28 | 2,07 | tyrosine kinase | protein tyrosine kinases |
| Q2_P32974 | 0,95 | 1,32 | tyrosine kinase, fyn |  |
| Q2_P29493 | 0,47 | 1,01 | tyrosine kinase, tec |  |
| Q2_P28077 | 0,38 | 0,84 | tyrosine kinase |  |
| Q2_P20704 | 1,09 | 1,88 | venus kinase receptor 2 (VKR2) |  |
| Q2_P39594 | 0,96 | 1,52 | insulin receptor |  |
|  | | | | |
| Q2_P38299 | 2,55 | 2,08 | eggshell protein chorion/  eggshell precursor protein | female-specific genes  female-specific genes |
| Q2_P25403 | 2,17 | 1,69 | similar to female-specific protein 800 (fs800) |  |
| Q2_P13217 | 2,08 | 1,78 | similar to female-specific protein 800 (fs800) |  |
| Q2_P22543 | 1,73 | 1,33 | major egg antigen (p40) |  |
| Q2_P00204 | 0,98 | 1,37 | major egg antigen (p40) |  |
| Q2_P25109 | 1,46 | 1,38 | ferritin |  |
| Q2_P26602 | 1,40 | 0,73 | ferritin light chain |  |
| Q2_P24734 | 1,91 | 0,69 | tyrosinase precursor |  |
|  | | | | |
| Q2_P37615 | 1,60 | 2,48 | early growth response protein | transcription factors |
| Q2_P02297 | 1,31 | 1,57 | early growth response protein |  |
| Q2_P02265 | 0,84 | 1,20 | early growth response protein |  |
| Q2_P29882 | 1,69 | 3,35 | zinc finger protein |  |
| Q2_P38843 | 2,82 | 2,06 |  |  |
| Q2_P01991 | 0,69 | 2,02 |  |  |
| Q2_P38877 | 0,83 | 1,35 |  |  |
| Q2_P33206 | 0,86 | 1,16 |  |  |
| Q2_P20084 | 0,89 | 1,07 |  |  |
| Q2_P05207 | 0,81 | 1,10 |  |  |
| Q2_P22175 | 0,71 | 1,12 |  |  |
| Q2_P19546 | 0,54 | 1,05 |  |  |
|  | | | | |
| Q2_P01955 | 1,36 | 1,94 | paramyosin | muscle proteins/  muscle contraction |
| Q2_P37137 | 0,64 | 1,07 | tropomyosin |  |
| Q2_P21901 | 0,25 | 0,75 | tropomyosin |  |
| Q2_P25195 | 0,86 | 1,73 | alpha-actinin |  |
| Q2_P26828 | 0,61 | 1,04 | alpha-actinin |  |
| Q2_P16525 | 0,80 | 0,47 | actin |  |
| Q2_P09566 | 0,62 | 1,64 | titin |  |
|  | | | | |
| Q2_P33844 | 1,28 | 1,88 | GTP binding protein | small GTPase signaling  small GTPase signaling |
| Q2_P20418 | 1,28 | 1,89 | Rab GTPase-activating protein |  |
| Q2_P20418 | 1,28 | 1,86 | Rab GTPase-activating protein |  |
| Q2_P01377 | 1,11 | 1,75 | Rap guanine nucleotide exchange factor |  |
| Q2_P14081 | 1,54 | 1,25 | growth factor receptor-bound protein, Ras-associated |  |
| Q2_P18010 | 0,88 | 1,54 | vav 2 guanine nucleotide exchange factor |  |
| Q2_P01285 | 0,63 | 1,78 | slit-robo rho gtpase activating protein |  |
| Q2_P18168 | 0,87 | 1,39 | rap1 |  |
| Q2_P04422 | 0,33 | 1,64 | guanine-nucleotide-exchange-factor |  |
| Q2_P09780 | 0,41 | 1,57 | G-protein signaling modulator |  |
| Q2_P19453 | 0,81 | 0,80 | developmentally regulated GTP-binding protein 1 (drg 1) |  |
| Q2_P29854 | 0,76 | 0,81 | Ras-associated |  |
| Q2_P01171 | 0,48 | 1,06 | Rho-GTPase-activating protein |  |
| Q2_P05839 | 0,59 | 0,94 | ras GTP exchange factor son of sevenless |  |
| Q2_P13509 | 0,78 | 0,70 | nucleolar GTP-binding protein |  |
| Q2_P29346 | 0,58 | 0,89 | Ras protein homologue |  |
| Q2_P01355 | 0,36 | 1,06 | rab GDP/GTP exchange factor |  |
| Q2_P24604 | 0,33 | 0,96 | guanine nucleotide-binding protein beta |  |
| Q2_P08655 | 0,30 | 0,91 | growth factor receptor-bound protein, Ras-associated |  |
| Q2_P20010 | 0,47 | 0,74 | GTP-binding protein alpha subunit gna |  |
| Q2_P33020 | 0,44 | 0,72 | GTP-binding protein era |  |
| Q2_P18918 | 0,37 | 0,58 | rcc1-related guanine nucleotide exchange factor |  |
|  | | | | |
| Q2_P31522 | 1,14 | 1,90 | heat shock protein 70 | heat shock proteins |
| Q2_P17905 | 0,34 | 1,25 | heat shock protein 70 |  |
| Q2_P26884 | 0,47 | 0,93 | heat shock protein 70 |  |
| Q2_P23191 | 0,62 | 0,76 | heat shock protein 70 |  |
| Q2_P23542 | 0,31 | 1,06 | heat shock protein 70 |  |
| Q2_P19624 | 1,13 | 1,39 | heat shock protein |  |
|  | | | | |
| Q2_P38119 | 0,52 | 1,76 | SWI/SNF complex-related | further signal transduction-associated proteins |
| Q2_P19849 | 0,38 | 0,62 | SWI/SNF-related chromatin binding protein |  |
| Q2_P17969 | 0,45 | 1,50 | Eps-15 |  |
| Q2_P14317 | 0,56 | 0,91 | immunophilin |  |
| Q2_P06918 | 0,22 | 1,13 | retinoic acid receptor RXR |  |
| Q2_P05666 | 0,49 | 0,48 | TGF-beta signal transducer Smad2 |  |
