# Supplementary material for: Imatinib Treatment Causes Substantial Transcriptional Changes in Adult Schistosoma mansoni In Vitro Exhibiting Pleiotropic Effects
Source: PLoS Negl Trop Dis. 2014 Jun 12;8(6):e2923. doi: 10.1371/journal.pntd.0002923 (PMC4055459; doi:10.1371/journal.pntd.0002923)
Supplement: Data S5 — List of selected genes down-regulated after Imatinib treatment (q = 0.1%). Besides the Gene ID number, relative transcript ratios are given for both time-points (24 h and 48 h) as well as annotations and functional categories. (DOCX) [file pntd.0002923.s005.docx]

Supplementary data 5

| **Gene ID** | **Ratio 24h** | **Ratio 48h** | **Annotation** | **Functional Category** |
| --- | --- | --- | --- | --- |
|  | | | | |
| Q2_P36194 | -2,38 | -5,47 | cathepsin B | cathepsins (proteases) |
| Q2_P35913 | -2,27 | -5,12 | cathepsin B |  |
| Q2_P27620 | -2,19 | -4,94 | cathepsin S |  |
| Q2_P26822 | -1,71 | -4,40 | cathepsin D2 |  |
| Q2_P26772 | -1,92 | -4,15 | cathepsin S |  |
| Q2_P38171 | -1,95 | -4,02 | cathepsin L-like |  |
| Q2_P19417 | -1,80 | -4,13 | cathepsin L |  |
| Q2_P06971 | -1,96 | -3,95 | cathepsin B-like |  |
| Q2_P09480 | -2,14 | -3,41 | cathepsin A-like |  |
| Q2_P25384 | -0,73 | -1,43 | precursor cathepsin C |  |
| Q2_P40354 | -0,52 | -1,47 | preproprotein cathepsin D - like |  |
| Q2_P41528 | -0,46 | -1,46 | preproprotein cathepsin D - like |  |
|  | | | | |
| Q2_P17916 | -1,66 | -3,95 | hemoglobinase (C13 family) |  |
|  | | | | |
| Q2_P12458 | -2,47 | -4,50 | scavenger receptor class B type-2 | receptors recognizing modified low-density lipoproteins (LDL); participate in the removal of many foreign substances and waste materials |
| Q2_P05178 | -2,25 | -3,80 | scavenger receptor class A-like |  |
| Q2_P19292 | -1,72 | -1,95 | CD36-like class B scavenger receptor |  |
| Q2_P05714 | -1,06 | -1,94 | macrophage scavenger receptor-related |  |
| Q2_P26539 | -0,84 | -0,94 | low-density lipoprotein receptor (ldl) |  |
|  | | | | |
| Q2_P17894 | -2,08 | -4,33 | venom allergen-like (VAL) 7 protein | VAL proteins, also known as sperm-coating proteins (SCP) |
| Q2_P12337 | -1,68 | -2,95 | venom allergen-like (VAL) 10 protein |  |
| Q2_P05412 | -0,40 | -2,27 | venom allergen-like (VAL) 8 protein |  |
|  | | | | |
| Q2_P05789 | -2,24 | -3,80 | metabotropic glutamate receptor | glutamate receptors/ transporters |
| Q2_P32236 | -1,99 | -2,78 | glutamate receptor AMPA |  |
| Q2_P00256 | -0,64 | -2,60 | glutamate receptor - like |  |
| Q2_P05303 | -1,50 | -1,21 | high affinity glutamate transporter |  |
|  |  |  |  |  |
| Q2_P12112 | -0,84 | -1,46 | monocarboxylate transporter | transporters |
| Q2_P21189 | -0,47 | -1,21 | monocarboxylate transporter |  |
| Q2_P23677 | -0,68 | -0,52 | monocarboxylate transporter |  |
| Q2_P06215 | -0,41 | -1,33 | monocarboxylate transporter - like |  |
| Q2_P35953 | -0,45 | -1,52 | sodium/sialic acid cotransporter |  |
| Q2_P38288 | -0,54 | -1,09 | sodium/chloride dependent transporter |  |
| Q2_P26688 | -0,34 | -1,29 | glucose transport protein |  |
| Q2_P00980 | -0,56 | -0,92 | cationic amino acid transporter |  |
| Q2_P23161 | -0,63 | -0,77 | glycerol-3-phosphate transporter |  |
| Q2_P37672 | -0,44 | -0,85 | cation efflux family protein |  |
| Q2_P18169 | -0,25 | -1,04 | sugar transporter |  |
| Q2_P25781 | -0,37 | -0,89 | UDP-glucuronic acid/UDP-N-acetylgalactosamine transporter |  |
| Q2_P14106 | -0,59 | -0,65 | acetyl-CoA transporter |  |
| Q2_P28047 | -0,60 | -0,57 | divalent metal transporter DMT1B |  |
| Q2_P03159 | -0,35 | -0,82 | cation chloride cotransporter |  |
|  | | | | |
| Q2_P21532 | -1,50 | -3,84 | gynecophoral canal protein (GCP) | gynecophoral canal protein |
| Q2_P18901 | -0,18 | -2,30 | gynecophoral canal protein (GCP) |  |
|  | | | | |
| Q2_P34786 | -1,37 | -3,14 | cytoplasmic dynein light chain | dynein light chain; motor protein; transports various cellular cargo by walking along cytoskeletal microtubules towards the minus-end of the microtubule |
| Q2_P05814 | -0,48 | -1,37 | cytoplasmic dynein light chain |  |
| Q2_P40593 | -1,08 | -2,48 | dynein light chain |  |
| Q2_P24579 | -0,62 | -2,66 | dynein light chain |  |
| Q2_P27118 | -0,89 | -1,94 | dynein light chain |  |
| Q2_P27304 | -1,09 | -1,49 | dynein light chain |  |
| Q2_P38292 | -0,51 | -1,34 | dynein light chain |  |
| Q2_P27258 | -0,23 | -1,33 | dynein light chain |  |
| Q2_P05945 | -0,72 | -1,06 | dynein light chain |  |
| Q2_P09080 | -0,31 | -0,66 | dynein light chain |  |
|  | | | | |
| Q2_P19267 | -1,98 | -1,89 | tubulin subunit alpha | tubulin; form microtubules (dimers of α- and β-tubulin) |
| Q2_P29512 | -0,87 | -0,95 | alpha tubulin |  |
| Q2_P19938 | -0,94 | -1,22 | tubulin subunit beta |  |
| Q2_P02489 | -0,67 | -1,20 | tubulin alpha - like |  |
| Q2_P07149 | -0,64 | -0,60 | tubulin beta-2C chain |  |
|  | | | | |
| Q2_P11045 | -1,96 | -1,50 | multidrug resistance protein (MDR) | drug efflux |
| Q2_P24890 | -1,00 | -0,83 | multidrug resistance protein (MDR) |  |
| Q2_P06121 | -0,69 | -1,61 | drug efflux protein-related |  |
|  | | | | |
| Q2_P40180 | -1,28 | -1,84 | integrin alpha chain | transmembrane proteins / receptors |
| Q2_P07555 | -1,19 | -1,66 | tetraspanin |  |
| Q2_P05966 | -0,67 | -1,57 | tetraspanin |  |
| Q2_P05763 | -0,82 | -0,80 | rhodopsin-like orphan GPCR |  |
| Q2_P32430 | -0,47 | -1,09 | frizzled-like receptor |  |
| Q2_P06147 | -0,50 | -0,40 | frizzled-like receptor |  |
|  | | | | |
| Q2_P27016 | -1,54 | -2,69 | calcium-binding protein/calmodulin | calmodulin; calcium binding protein |
| Q2_P17374 | -1,01 | -2,54 | calmodulin-like |  |
| Q2_P20304 | -0,85 | -2,15 | calmodulin-like |  |
| Q2_P18476 | -0,78 | -2,41 | calmodulin |  |
| Q2_P25438 | -0,60 | -0,84 | calmodulin |  |
| Q2_P17488 | -0,68 | -0,35 | calmodulin |  |
| Q2_P04689 | -0,78 | -1,15 | calmodulin related calcium binding protein |  |
| Q2_P34213 | -0,85 | -0,71 | calmodulin-related |  |
| Q2_P23683 | -0,49 | -0,80 | calcineurin B subunit |  |
|  | | | | |
| Q2_P21460 | -0,68 | -1,55 | histone H2A | histone H2A; involved in the structure of chromatin in eukaryotic cells |
| Q2_P39591 | -0,42 | -1,33 | histone H2A |  |
| Q2_P07060 | -0,26 | -1,24 | histone H2A |  |
| Q2_P02503 | -0,45 | -0,69 | histone H2A |  |
| Q2_P40444 | -0,47 | -0,49 | histone H2A |  |
|  | | | | |
| Q2_P41332 | -0,20 | -1,10 | spermatogenesis associated 17-like | spermatogenesis / testis associated |
| Q2_P06030 | -0,29 | -0,89 | testis specific protein |  |
|  | | | | |
| Q2_P35602 | -0,30 | -2,25 | Nanos RNA binding domain | morphogen-binding |
| Q2_P05740 | -0,39 | -0,83 | Fak-like tyrosine kinase | signal transduction |
| Q2_P21794 | -0,47 | -0,59 | immunophilin FK506 binding protein FKBP12 | signal transduction-associated |
|  | | | | |
| Q2_P12670 | -1,38 | -0,81 | cadherin | cell adhesion |
